# Supplementary material for: The Isolation, Structure Elucidation and Bioactivity Study of Chilensosides A, A1, B, C, and D, Holostane Triterpene Di-, Tri- and Tetrasulfated Pentaosides from the Sea Cucumber Paracaudina chilensis (Caudinidae, Molpadida)
Source: Molecules. 2022 Nov 7;27(21):7655. doi: 10.3390/molecules27217655 (PMC9658831; doi:10.3390/molecules27217655)
Supplement: Supplementary file 1 [file molecules-27-07655-s001.zip › molecules-1979839-supplementary.pdf]

Supplementary data content page

Title: The Isolation, Structure Elucidation and Bioactivity Study of Chilensosides A, A_1, B, C, and D, Holostane Triterpene Di-, Tri- and Tetrasulfated Pentaosides from the Sea Cucumber *Paracaudina chilensis* (Caudinidae, Molpadida)

Authors: AlexandraS.Silchenko *, Sergey A. Avilov, Pelageya V. Andrijaschenko, Roman S. Popov, Ekaterina A. Chingizova, Boris B. Grebnev, Anton B. Rasin and Vladimir I. Kalinin

Address: *G.B. Elyakov Pacific Institute of Bioorganic Chemistry, Far Eastern Branch of Russian Academy of Sciences, Pr. 100-let Vladivostoka 159, 690022 Vladivostok, Russia*

Correspondence: silchenko_als@piboc.dvo.ru; Tel.: +7-423-231-1168

Content:

Figure S1. The ^{13}C NMR (176.04 MHz) spectrum of chilensoside A (**1**) in C_5D_5N/D_2O (4/1)

Figure S2. The ^1H NMR (700.13 MHz) spectrum of chilensoside A (**1**) in C_5D_5N/D_2O (4/1)

Figure S3. The COSY (700.13 MHz) spectrum of chilensoside A (**1**) in C_5D_5N/D_2O (4/1)

Figure S4. The HSQC (700.13 MHz) spectrum of chilensoside A (**1**) in C_5D_5N/D_2O (4/1)

Figure S5. The ROESY (700.13 MHz) spectrum of chilensoside A (**1**) in C_5D_5N/D_2O (4/1)

Figure S6. The HMBC (700.13 MHz) spectrum of chilensoside A (**1**) in C_5D_5N/D_2O (4/1)

Figure S7. 1 D TOCSY (700.13 MHz) spectra of Xyl1, Qui2, Glc3, Glc4, MeGlc5 of chilensoside A (**1**) in C_5D_5N/D_2O (4/1)

Figure S8. HR-ESI-MS and ESI-MS/MS spectra of chilensoside A (**1**)

Table S1.^{13}C and ^1H NMR chemical shifts, HMBC and ROESY correlations of carbohydrate moiety of chilensosideA_1 (**2**)

Figure S9. The ^{13}C NMR (125.67 MHz) spectrum of chilensoside A_1 (**2**) in C_5D_5N/D_2O (4/1)

Figure S10. The ^1H NMR (500.12 MHz) spectrum of chilensoside A_1 (**2**) in C_5D_5N/D_2O (4/1)

Figure S11. The COSY (500.12 MHz) spectrum of chilensoside A_1 (**2**) in C_5D_5N/D_2O (4/1)

Figure S12. The HSQC (500.12 MHz) spectrum of chilensoside A_1 (**2**) in C_5D_5N/D_2O (4/1)

Figure S13. The HMBC (500.12 MHz) spectrum of chilensoside A_1 (**2**) in C_5D_5N/D_2O (4/1)

Figure S14. The ROESY (500.12 MHz) spectrum of chilensoside A_1 (**2**) in C_5D_5N/D_2O (4/1)

Figure S15. 1D TOCSY (500.12 MHz) spectra of Xyl1, Qui2, Glc3, Glc4, MeGlc5 of chilensoside A_1 (**2**) in C_5D_5N/D_2O (4/1)

Figure S16. HR-ESI-MS and ESI-MS/MS spectra of chilensoside A_1 (**2**)

Table S2. ^{13}C and ^{1}H NMR chemical shifts, HMBC and ROESY correlations of the aglycone part of chilensoside B (**3**)

Figure S17. The ^{13}C NMR (176.04 MHz) spectrum of chilensoside B (**3**) in C_5D_5N/D_2O (4/1)

Figure S18. The ^{1}H NMR (700.13 MHz) spectrum of chilensoside B (**3**) in C_5D_5N/D_2O (4/1)

Figure S19. The COSY (700.13 MHz) spectrum of chilensoside B (**3**) in C_5D_5N/D_2O (4/1)

Figure S20. The HSQC (700.13 MHz) spectrum of chilensoside B (**3**) in C_5D_5N/D_2O (4/1)

Figure S21. The HMBC (700.13 MHz) spectrum of chilensoside B (**3**) in C_5D_5N/D_2O (4/1)

Figure S22. The ROESY (700.13 MHz) spectrum of chilensoside B (**3**) in C_5D_5N/D_2O (4/1)

Figure S23. 1 D TOCSY (700.13 MHz) spectra of Xyl1, Qui2, Glc3, Glc4, MeGlc5 of chilensoside B (**3**) in C_5D_5N/D_2O (4/1)

Figure S24. HR-ESI-MS and ESI-MS/MS spectra of chilensoside B (**3**)

Table S3. ^{13}C and ^{1}H NMR chemical shifts, HMBC and ROESY correlations of the aglycone part of chilensoside C (**4**)

Figure S25. The ^{13}C NMR (176.04 MHz) spectrum of chilensoside C (**4**) in C_5D_5N/D_2O (4/1)

Figure S26. The ^{1}H NMR (700.13 MHz) spectrum of chilensoside C (**4**) in C_5D_5N/D_2O (4/1)

Figure S27. The COSY (700.13 MHz) spectrum of chilensoside C (**4**) in C_5D_5N/D_2O (4/1)

Figure S28. The HSQC (700.13 MHz) spectrum of chilensoside C (**4**) in C_5D_5N/D_2O (4/1)

Figure S29. The ROESY (700.13 MHz) spectrum of chilensoside C (**4**) in C_5D_5N/D_2O (4/1)

Figure S30. The HMBC (700.13 MHz) spectrum of chilensoside C (**4**) in C_5D_5N/D_2O (4/1)

Figure S31. 1D TOCSY (700.13 MHz) spectra of Xyl1, Qui2, Glc3, Glc4, MeGlc5 of chilensoside C (**4**) in C_5D_5N/D_2O (4/1)

Figure S32. HR-ESI-MS and ESI-MS/MS spectra of chilensoside C (**4**)

Table S4. ^{13}C and ^{1}H NMR chemical shifts, HMBC and ROESY correlations of the aglycone part of chilensoside D (**5**)

Figure S33. The ^{13}C NMR (125.67 MHz) spectrum of chilensoside D (**5**) in C_5D_5N/D_2O (4/1)

Figure S34. The ^{1}H NMR (500.12 MHz) spectrum of chilensoside D (**5**) in C_5D_5N/D_2O (4/1)

Figure S35. The COSY (500.12 MHz) spectrum of chilensoside D (**5**) in C_5D_5N/D_2O (4/1)

Figure S36. The HSQC (500.12 MHz) spectrum of chilensoside D (**5**) in C_5D_5N/D_2O (4/1)

Figure S37. The ROESY (500.12 MHz) spectrum of chilensoside D (**5**) in C_5D_5N/D_2O (4/1)

Figure S38. The HMBC (500.12 MHz) spectrum of chilensoside D (**5**) in C_5D_5N/D_2O (4/1)

Figure S39. 1D TOCSY (500.12 MHz) spectra of Xyl1, Qui2, Glc3, Glc4, MeGlc5 of chilensoside D (**5**) in C_5D_5N/D_2O (4/1)

Figure S40. HR-ESI-MS and ESI-MS/MS spectra of chilensoside D (**5**)

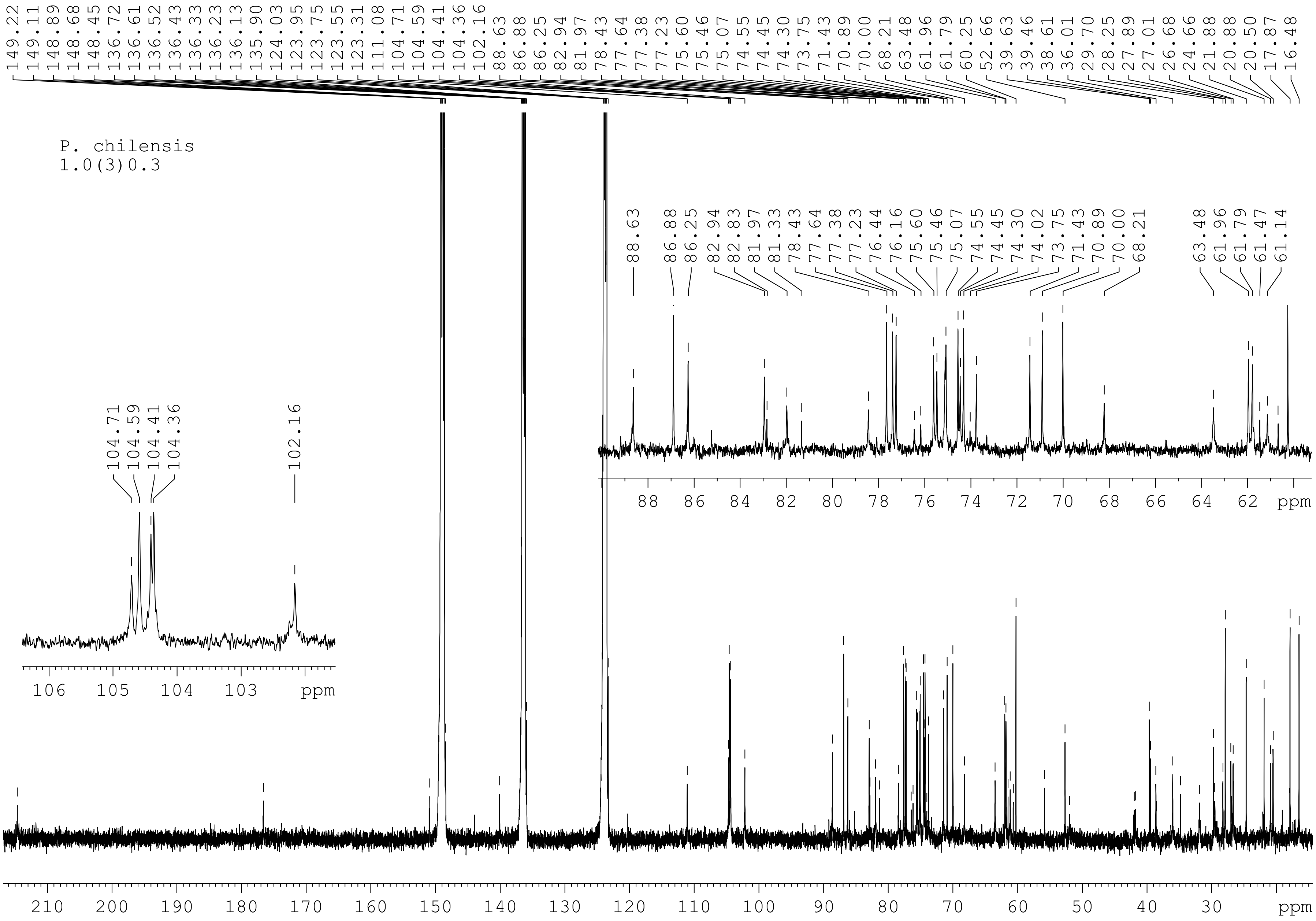

Figure S1. The ^{13}C NMR (176.04 MHz)spectrum of chilensosideA (**1**) in C_5D_5N/D_2O (4/1)

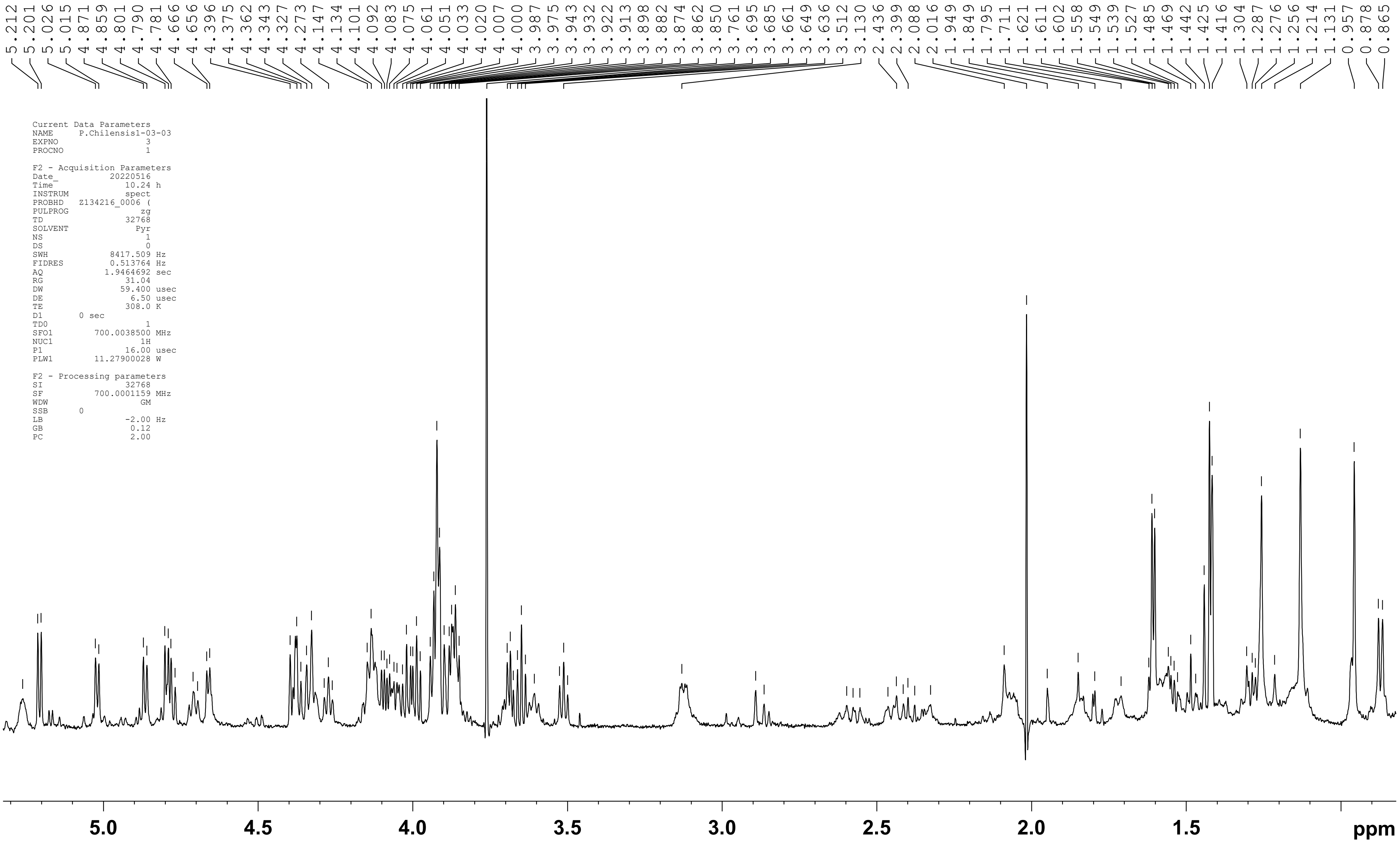

Figure S2. The ¹H NMR (700.13 MHz) spectrum of chilensoside A (**1**) in C_5D_5N/D_2O (4/1)

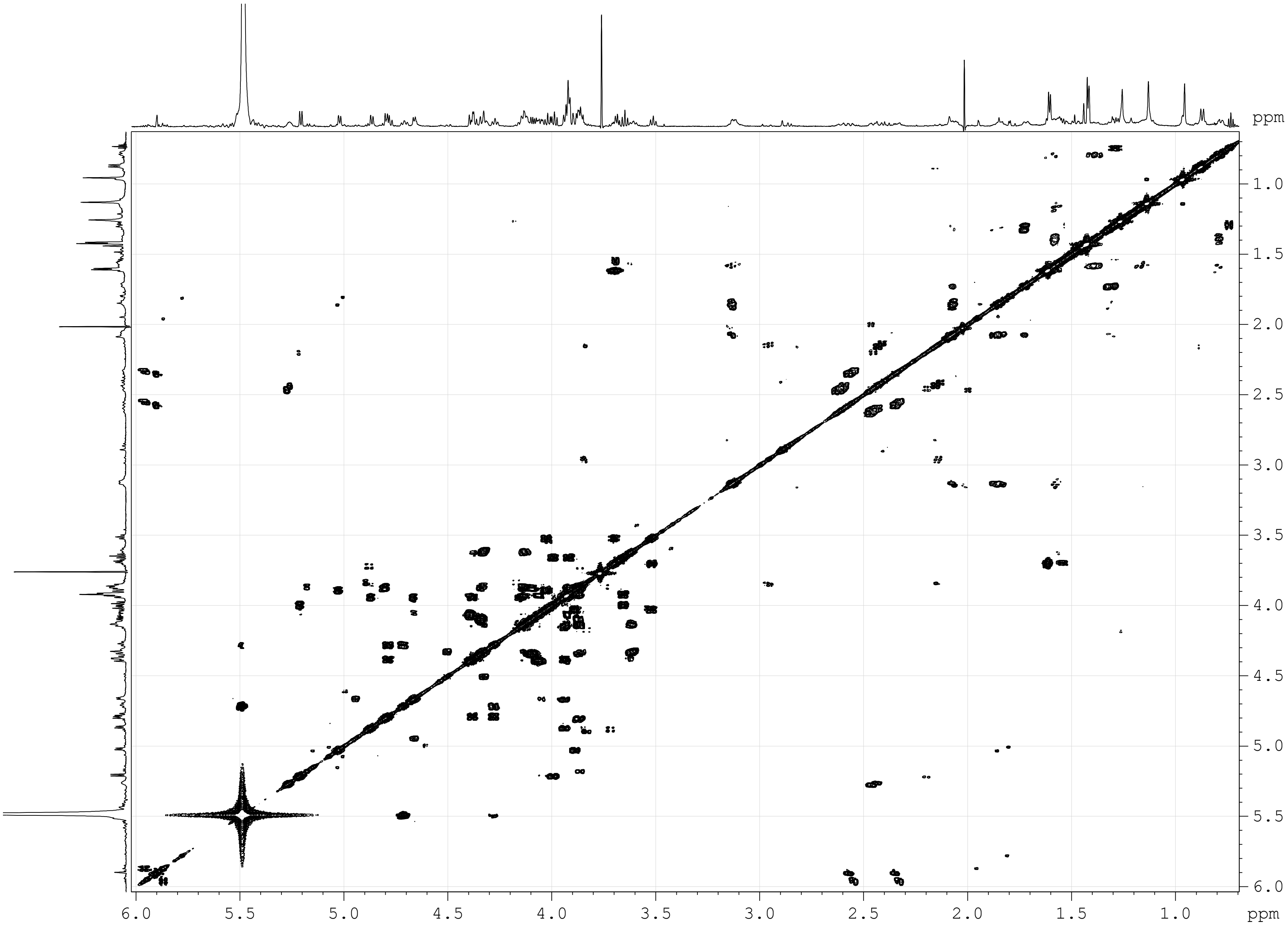

Figure S3. The COSY (700.13 MHz) spectrum of chilensoside A (**1**) in C_5D_5N/D_2O (4/1)

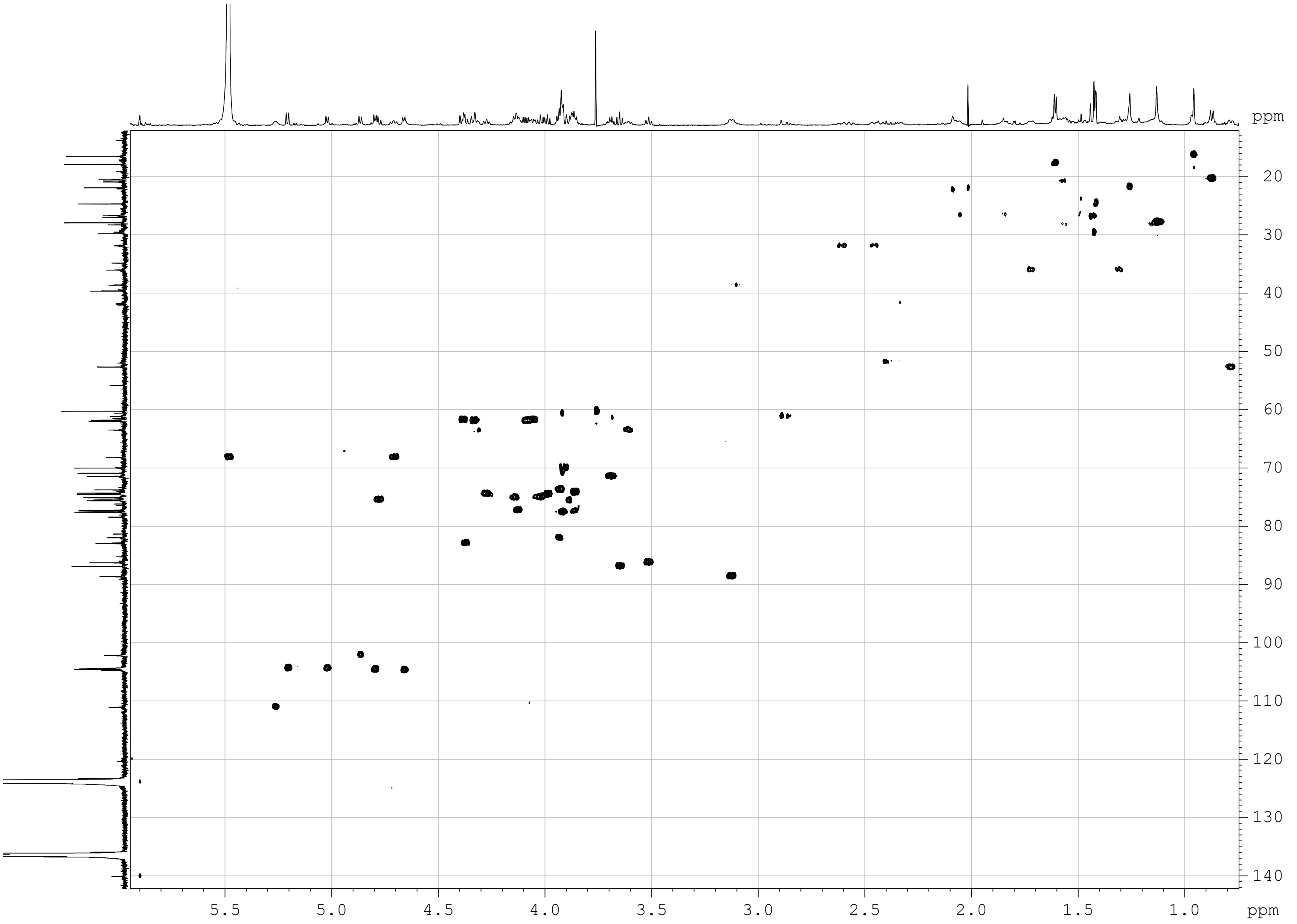

Figure S4. The HSQC (700.13 MHz) spectrum of chilensoside A (**1**) in C_5D_5N/D_2O (4/1)

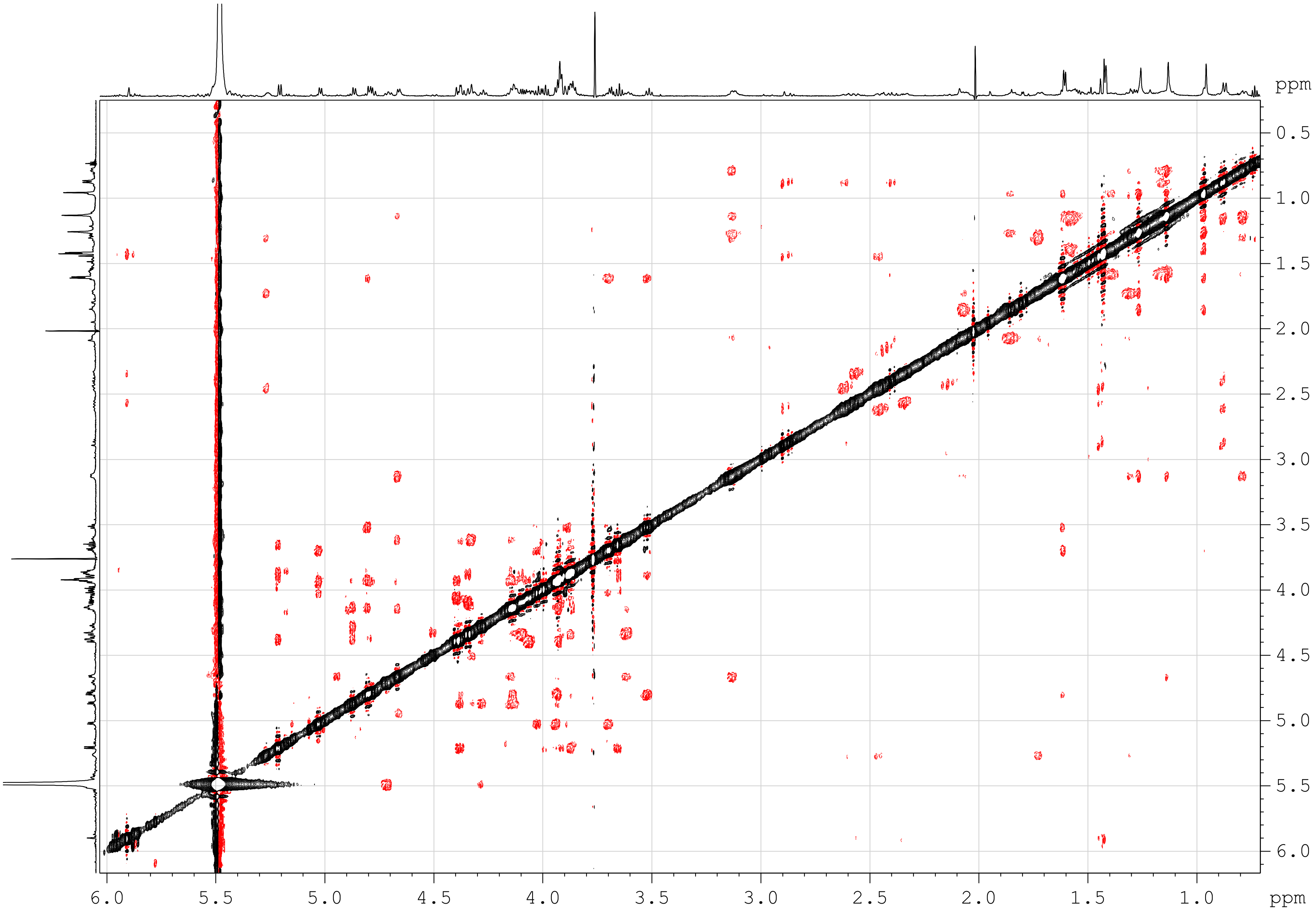

Figure S5. The ROESY (700.13 MHz) spectrum of chilensoside A (**1**) in C_5D_5N/D_2O (4/1)

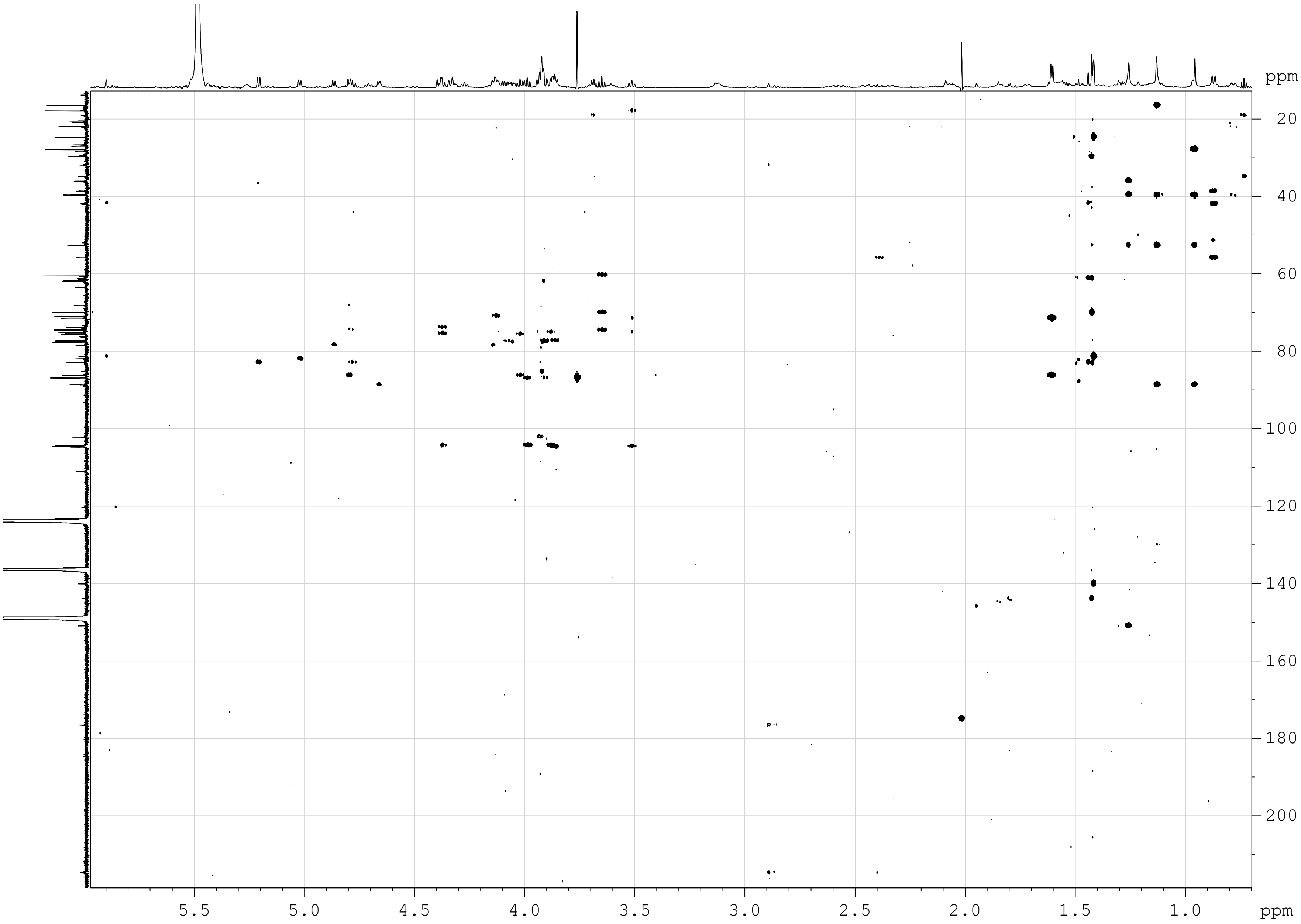

Figure S6. The HMBC (700.13 MHz) spectrum of chilensoside A (**1**) in C_5D_5N/D_2O (4/1)

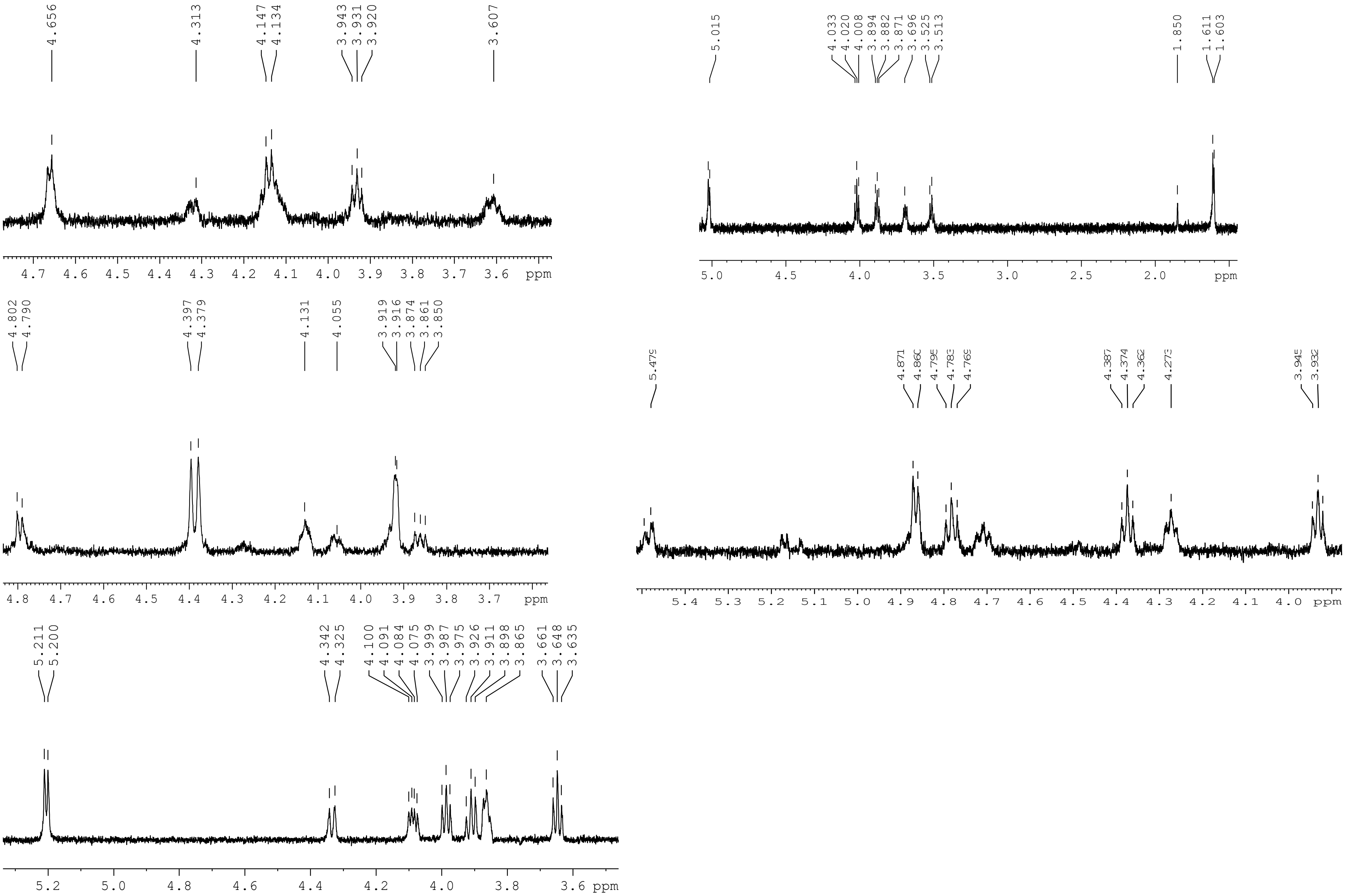

Figure S7. 1 D TOCSY (700.13 MHz) spectra of Xyl1, Qui2, Glc3, Glc4, MeGlc5 of chilensoside A (**1**) in C_5D_5N/D_2O (4/1)

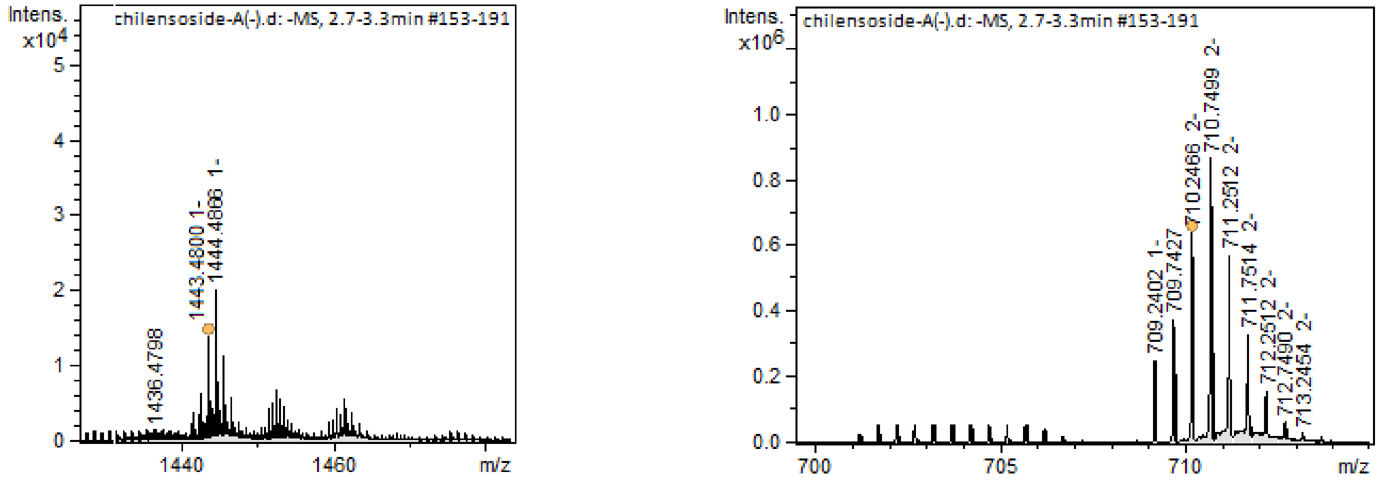

* The isotopic composition in the HR-ESI-MS of chilensosides A–B (**1**–**3**), where the ion peak of $[M_{Na}+1]^{-}$ is more intensive, than that of $[M_{Na}]^{-}$ is explained by the easy exchange of the protons at C-15, adjacent to 16-oxo-group, to deuterium during the forced long-term storage of the samples in C_5D_5N/D_2O for the registration of the NMR spectra.

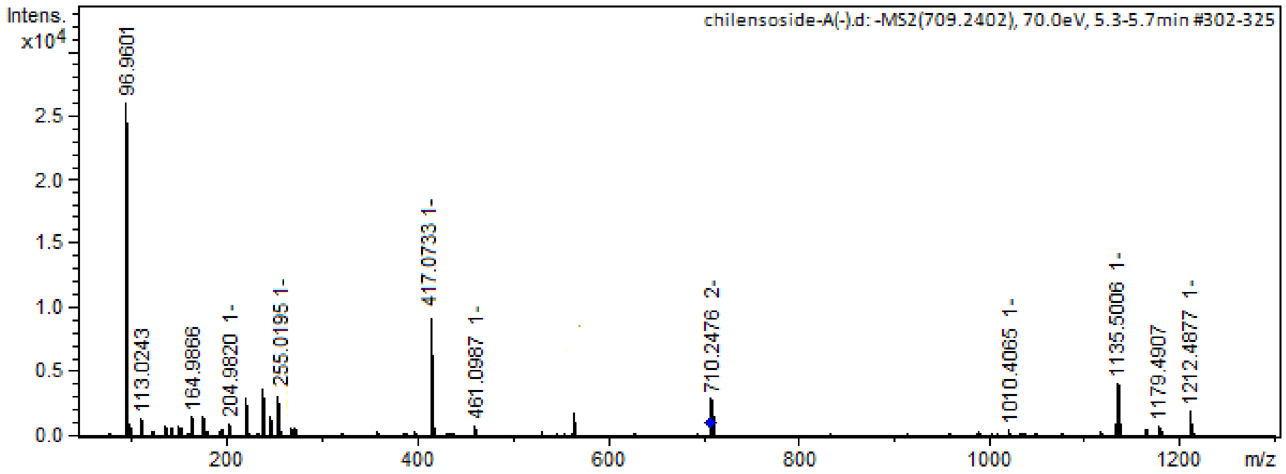

Figure S8. HR-ESI-MS and ESI-MS/MS spectra of chilensoside A (**1**)

Table S1: ^{13}C and ^{1}H NMR chemical shifts and HMBC and ROESY correlations of carbohydrate moiety of chilensoside A_1 (**2**)

Atom	$\delta_C$mult. [a, b, c]	$\delta_H$mult. (*J* in Hz) [d]	HMBC	ROESY
Xyl1 (1→C-3)				
1	104.7CH	4.66d (6.6)	C: 3	H-3; H-3, 5 Xyl1
2	**82.1**CH	3.90t (8.0)	C: 1 Qui2	H-1Qui2
3	75.1CH	4.12t (8.0)		
4	**78.8** CH	4.10 m		H-1 Glc4
5	63.5 CH_2	4.32m		
		3.60m		H-1 Xyl1
Qui2 (1→2Xyl1)				
1	104.4 CH	4.98 d (8.2)	C: 2 Xyl1	H-2 Xyl1; H-3, 5 Qui2
2	75.6 CH	3.89 t (8.2)		H-4 Qui2
3	74.9 CH	4.04 t (8.2)		H-1, 5 Qui2
4	**86.1** CH	3.50 t (8.2)	C: 1 Glc3	H-1 Glc3
5	71.5 CH	3.68 dd (5.7; 8.2)		H-1 Qui2
6	17.8 CH_3	1.60 d (5.7)	C: 4, 5 Qui2	H-4 Qui2
Glc3 (1→4Qui2)				
1	104.6 CH	4.79 d (8.5)	C: 4 Qui2	H-4 Qui2; H-3, 5Glc3
2	74.4 CH	3.85 t (8.5)		
3	77.2 CH	4.13 t (8.5)		H-1, 5Glc3
4	70.9 CH	3.92 m		H-6 Glc3
5	77.7 CH	3.91 m		H-1 Glc3
6	61.7CH_2	4.39d (11.6)		
		4.05 dd (5.5; 11.6)		
Glc4(1→4Xyl1)				
1	102.3 CH	4.85 d (7.5)	C: 4 Xyl1	H-4 Xyl1; H-3, 5 Glc4
2	73.7 CH	3.93 t (8.6)		
3	**83.0** CH	4.37 t (8.6)	C: 1 MeGlc5	H-1 MeGlc5; H-1 Glc4
4	*75.5* CH	4.78 t (8.6)		
5	74.3 CH	4.26 t (9.7)		H-1 Glc4
6	*68.3* CH_2	5.50 m		
		4.71 dd (9.7; 11.8)		
MeGlc5(1→3Glc4)				
1	104.4 CH	5.20 d (7.7)	C: 3 Glc4	H-3 Glc4; H-3,5 MeGlc5
2	74.6 CH	3.99 t (8.7)		
3	86.9 CH	3.65 t (8.7)		H-1 Me Glc5
4	70.0 CH	3.91 t (8.7)		
5	77.4 CH	3.86 t (8.7)		H-1 MeGlc5
6	62.0 CH_2	4.34 d (10.6)		
		4.09 dd (6.8; 11.6)		H-4 MeGlc5
OMe	60.3 CH_3	3.76 s	C: 3 MeGlc5	

[a] Recorded at 125.67 MHz in C_5D_5N/D_2O (4/1). [b] Bold = interglycosidic positions. [c] Italic = sulfate position. [d] Recorded at 500.12 MHz in C_5D_5N/D_2O (4/1). Multiplicity by 1D TOCSY.

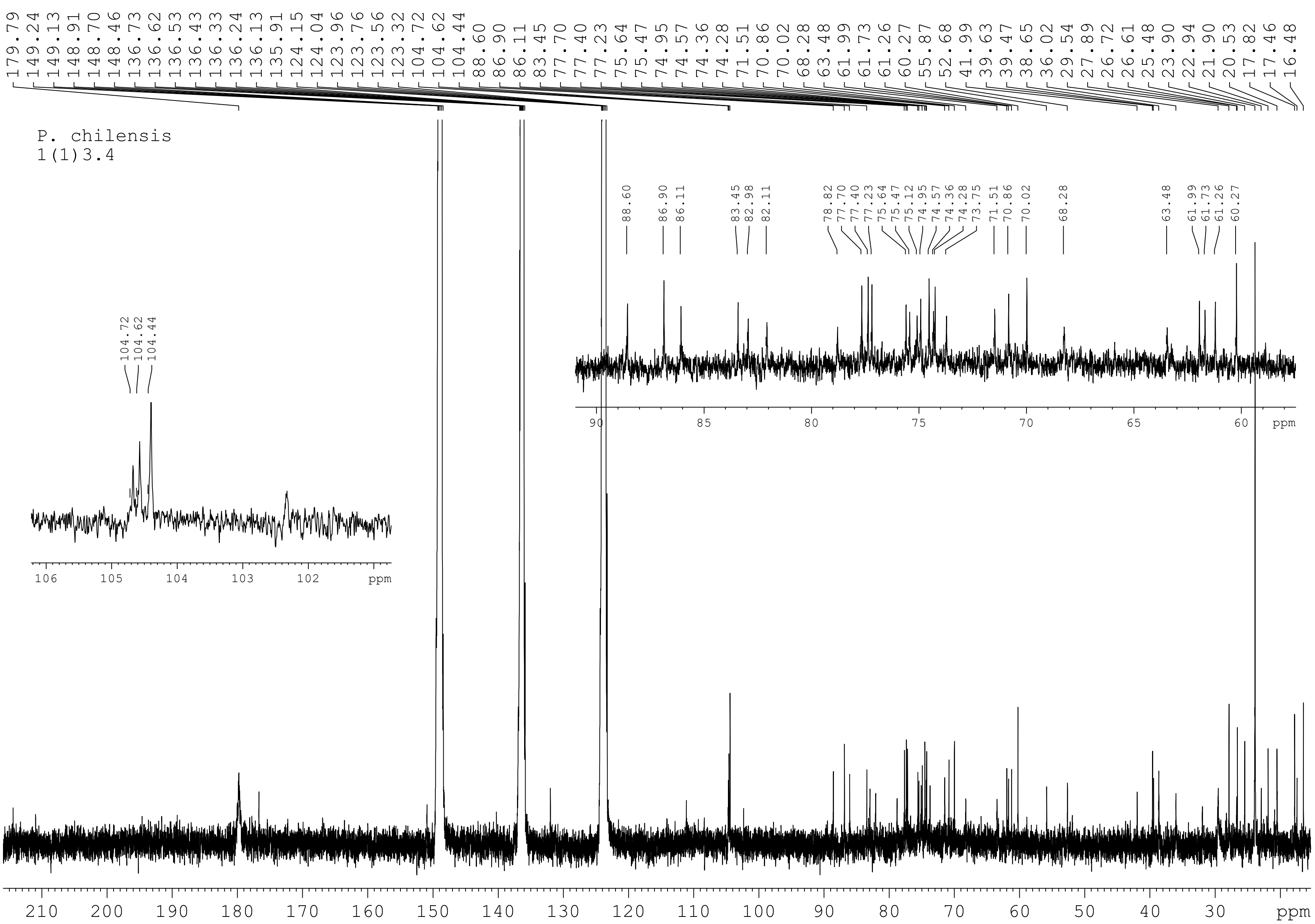

Figure S9. The ^{13}C NMR (125.67 MHz) spectrum of chilensoside A$_1$ (**2**) in C_5D_5N/D_2O (4/1)

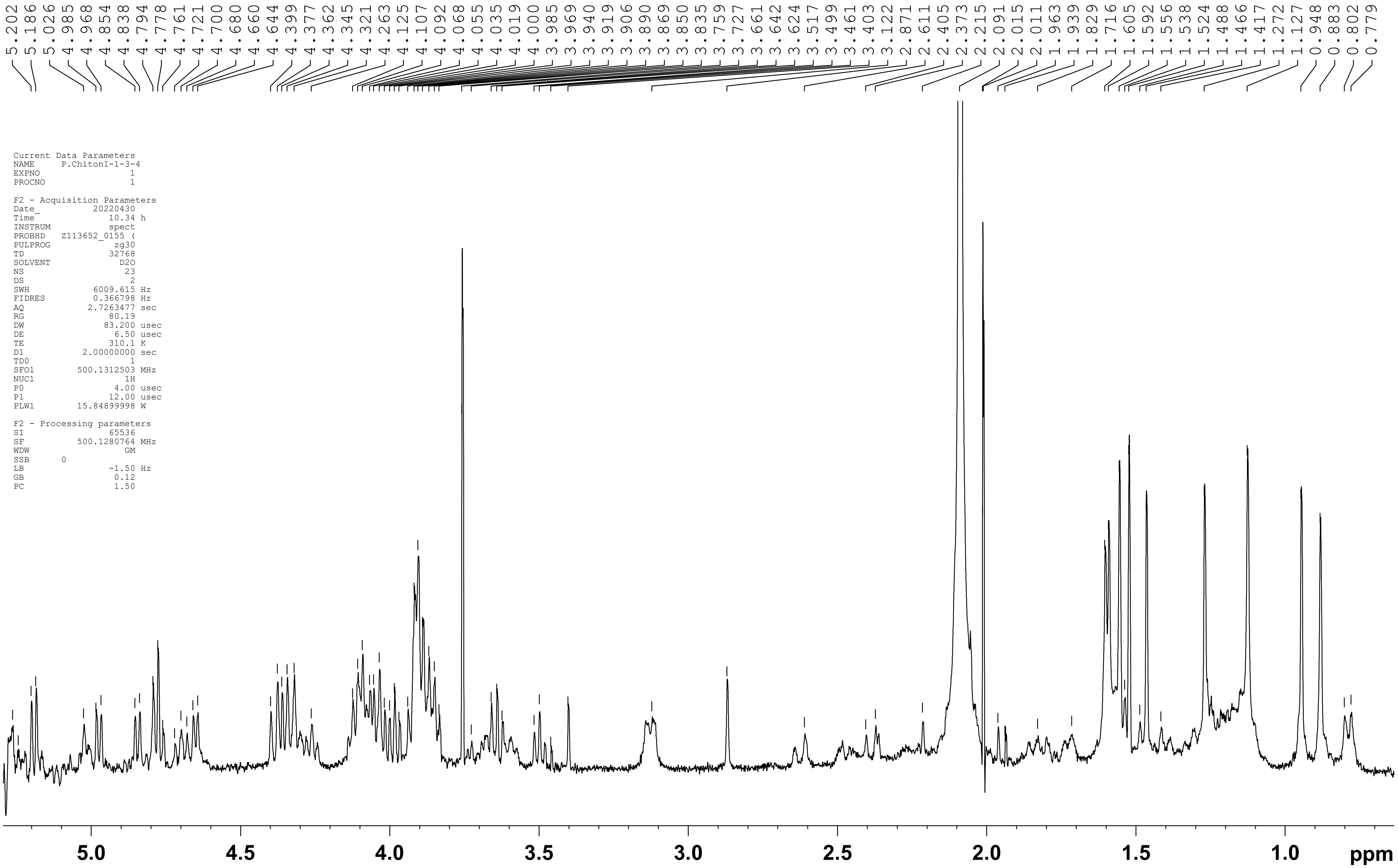

Figure S10. The ^{1}H NMR (500.12 MHz) spectrum of chilensoside A_1 (**2**) in C_5D_5N/D_2O (4/1)

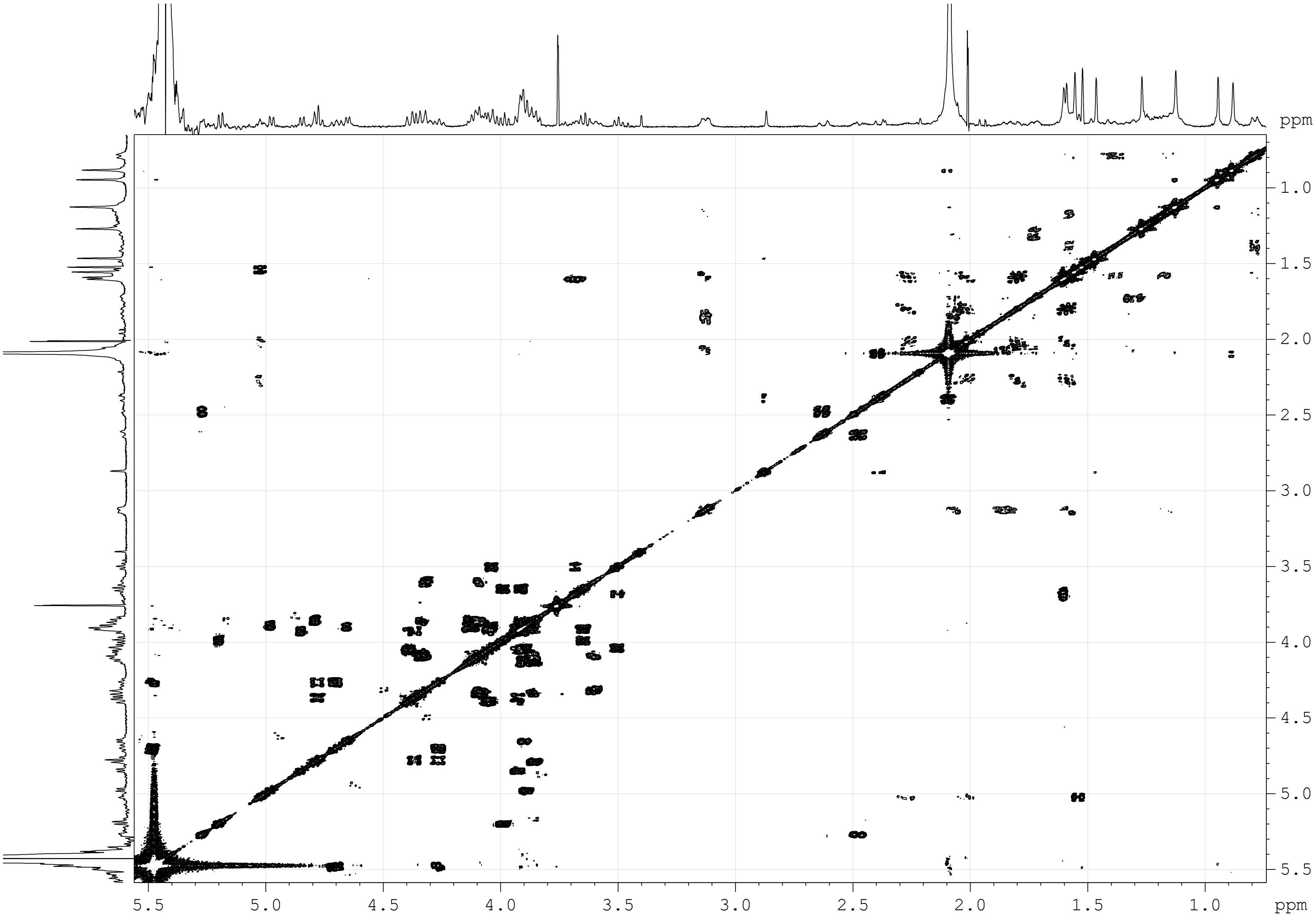

Figure S11. The COSY (500.12 MHz) spectrum of chilensoside A1 (**2**) in C_5D_5N/D_2O (4/1)

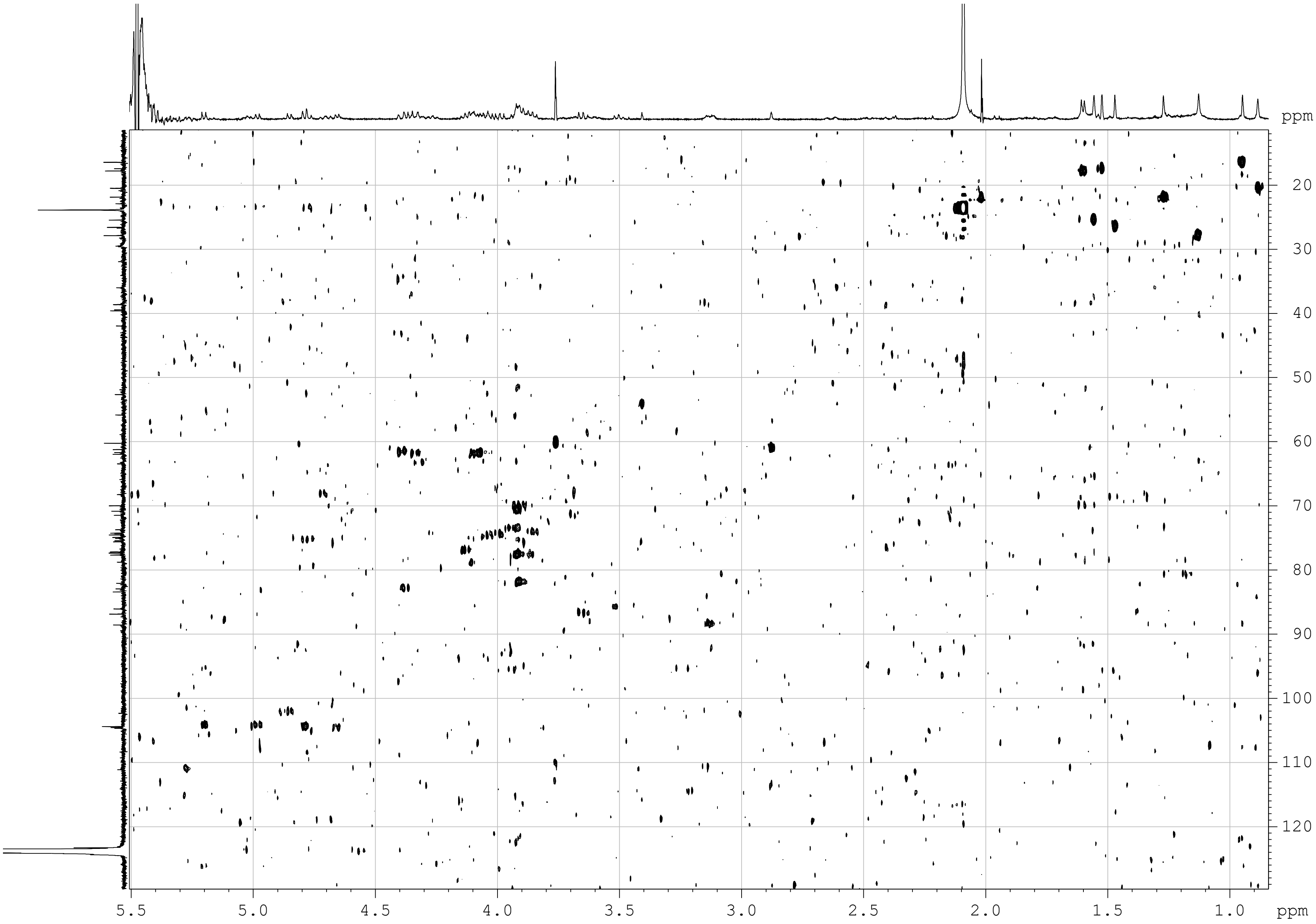

Figure S12. The HSQC (500.12 MHz) spectrum of chilensoside A1 (**2**) in C_5D_5N/D_2O (4/1)

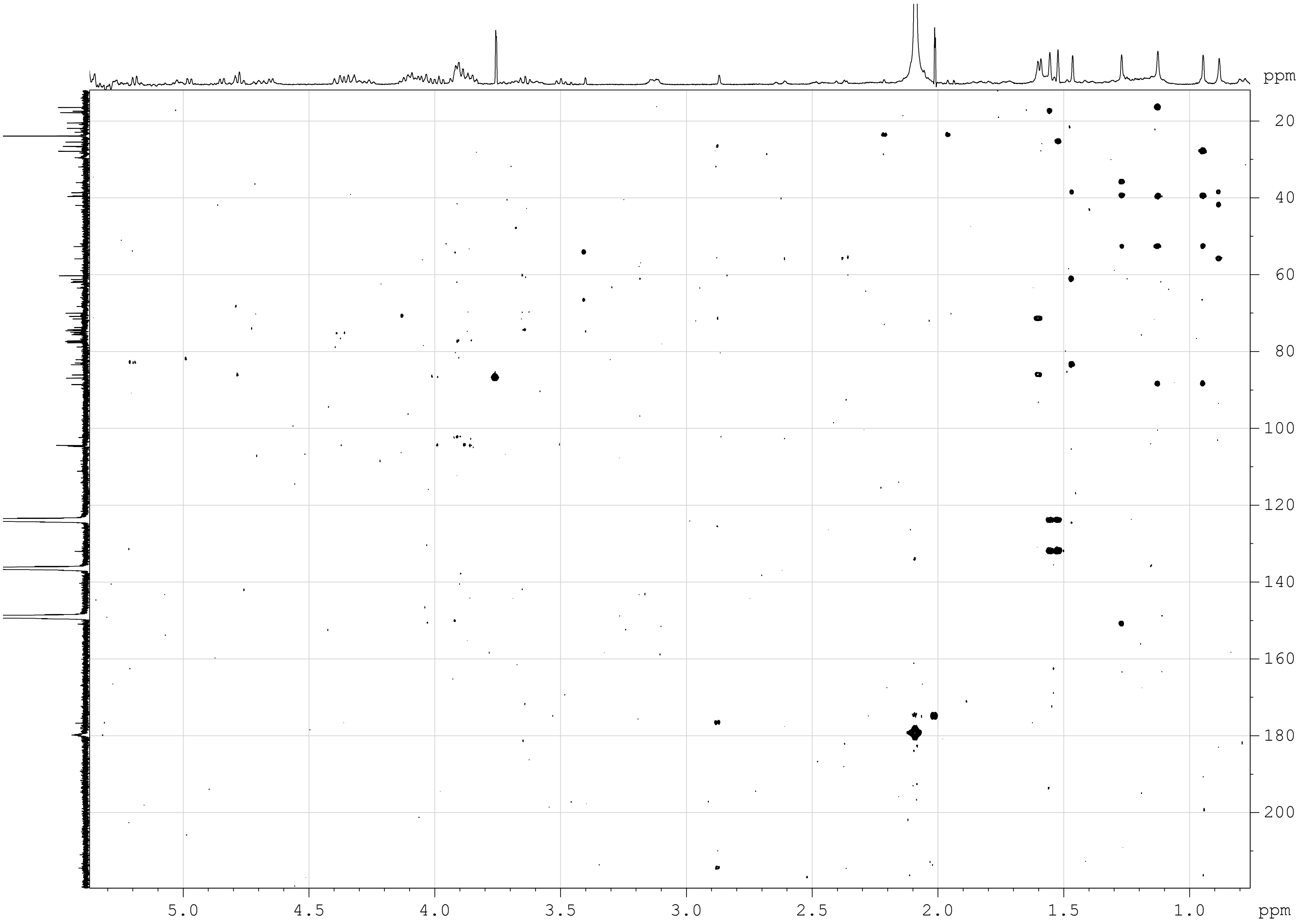

Figure S13. The HMBC (500.12 MHz) spectrum of chilensoside A1 (**2**) in C_5D_5N/D_2O (4/1)

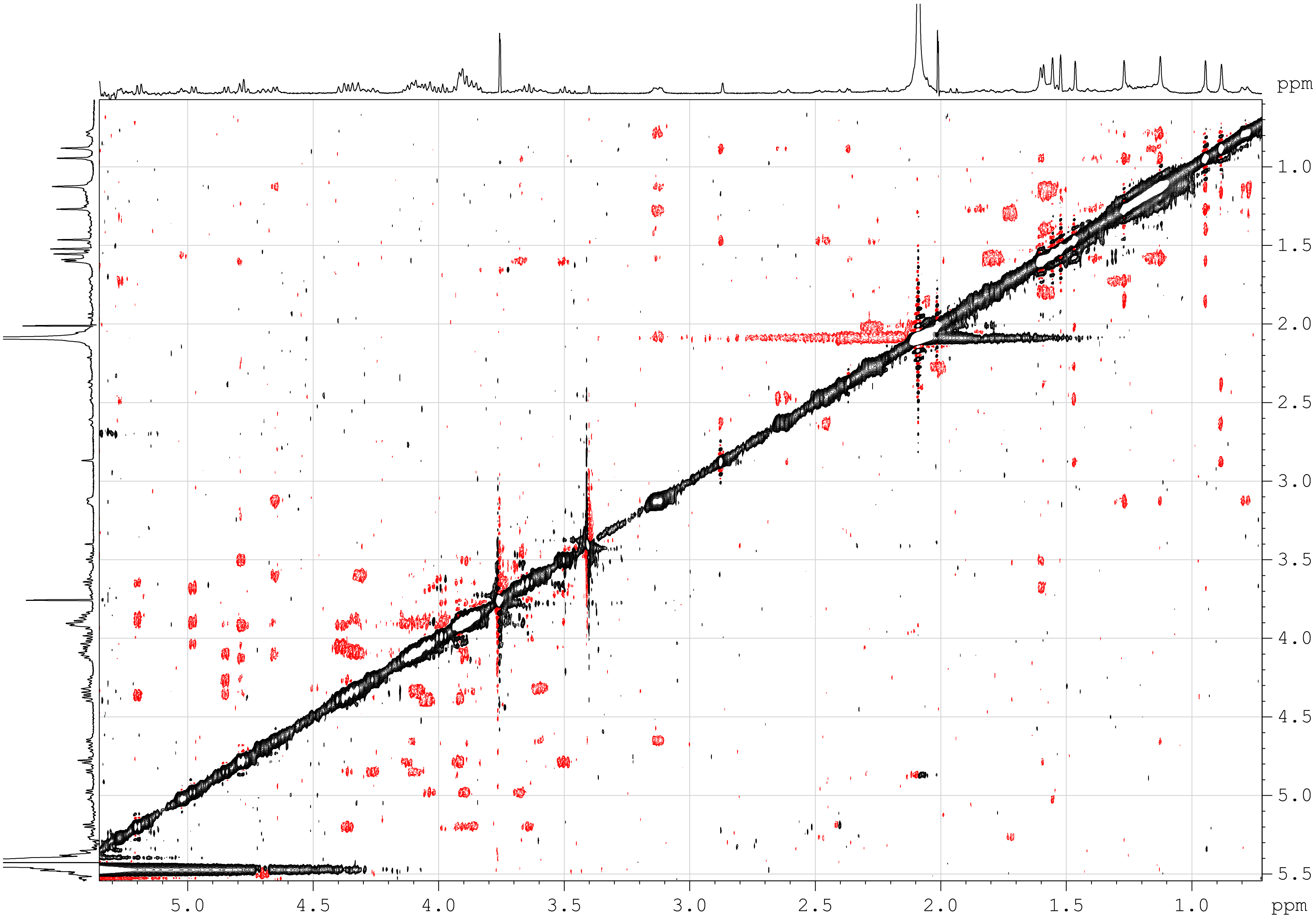

Figure S14. The ROESY (500.12 MHz) spectrum of chilensoside A1 (**2**) in C_5D_5N/D_2O (4/1)

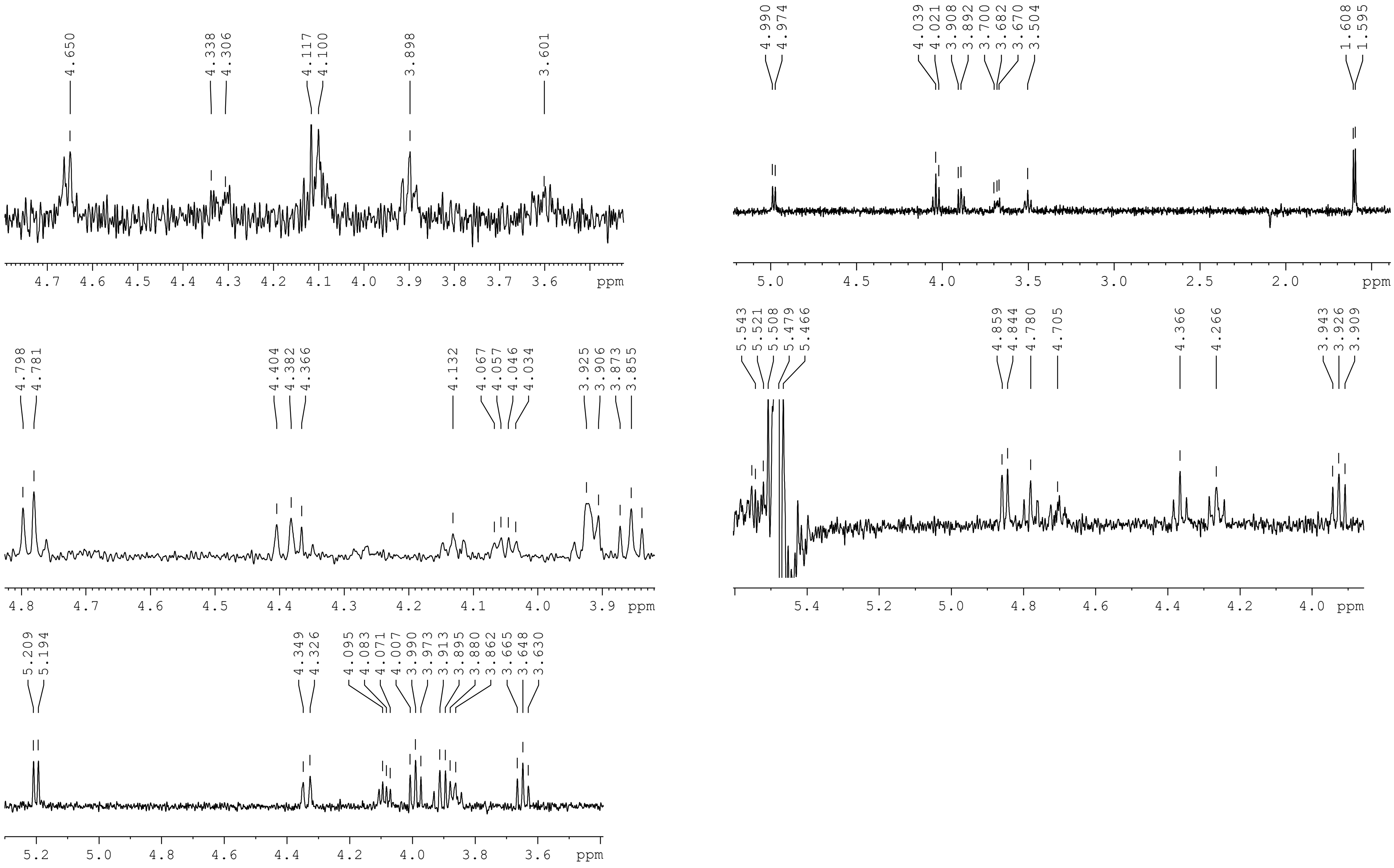

Figure S15. 1D TOCSY (500.12 MHz) spectra of Xyl1, Qui2, Glc3, Glc4, MeGlc5 of chilensoside A1 (**2**) in C_5D_5N/D_2O (4/1)

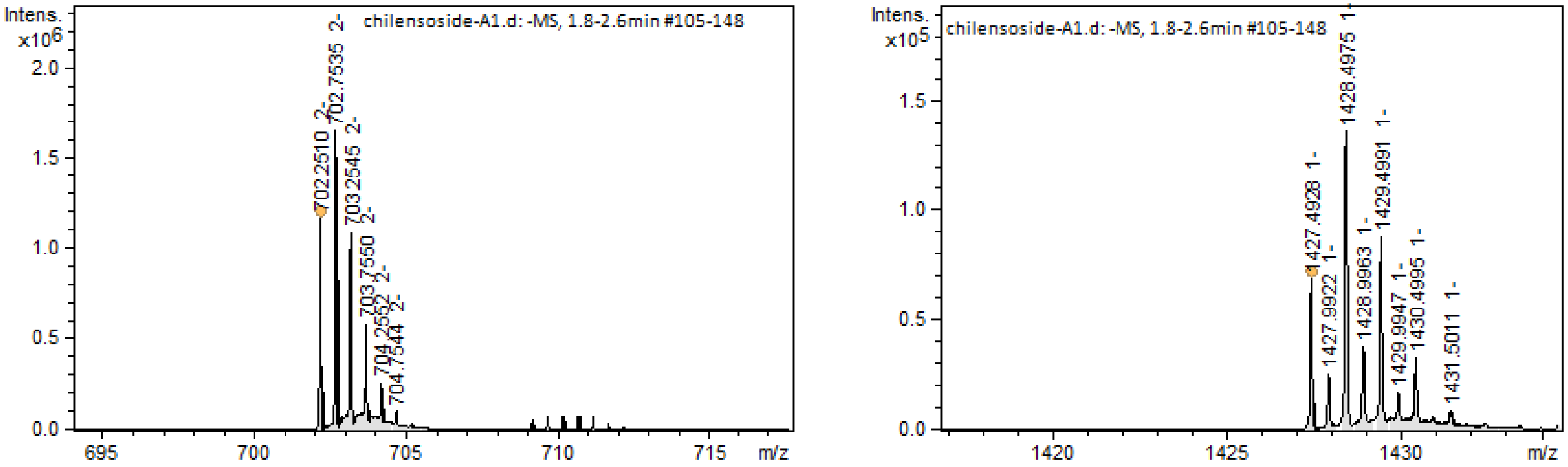

∗The isotopic composition in the HR-ESI-MS of chilensosides A–B (**1–3**), where the ion peak of $[M_{Na}+1]^-$ is more intensive, than that of $[M_{Na}]^-$ is explained by the easy exchange of the protons at C-15, adjacent to 16-oxo-group, to deuterium during the forced long-term storage of the samples in C_5D_5N/D_2O for the registration of the NMR spectra.

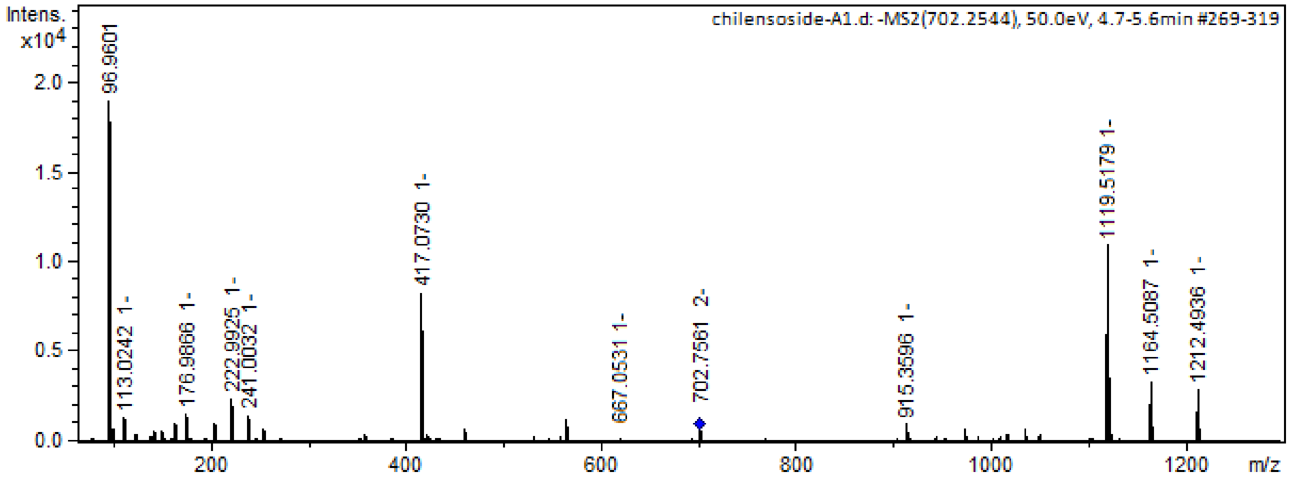

Figure S16. HR-ESI-MS and ESI-MS/MS spectra of chilensoside A_1 (**2**)

Table S2. ^{13}C and ^{1}H NMR chemical shifts, HMBC and ROESY correlations of the aglycone moiety of chilensoside B (**3**).

Position	δ_C mult.[a]	δ_H mult. (*J* in Hz)[b]	HMBC	ROESY
1	36.0 CH_2	1.73 m		H-11
		1.32 m		H-3
2	26.6 CH_2	2.07 m		
		1.86 m		H-19, H-30
3	88.6 CH	3.13 dd (4.7; 11.8)		H-1, H-5, H-31, H1-Xyl1
4	39.4 C			
5	52.6 CH	0.79 brd (11.8)	C: 4, 19, 30	H-1, H-3, H-7
6	20.8 CH_2	1.59 m		
		1.39 m		H-8, H-30
7	28.2 CH_2	1.58 m		H-15
		1.16 m		H-5, H-32
8	38.6 CH	3.13 m		H-6
9	151.1 C			
10	39.6 C			
11	111.3 CH	5.28 brs	C: 10, 13	H-1
12	31.9 CH_2	2.64 brd (16.5)	C: 11, 18	H-17
		2.48 dd (5.9; 16.5)	C: 11, 14	
13	55.8 C			
14	41.9 C			
15	51.8 CH_2	2.40 d (16.0)	C: 13, 16, 17, 32	
		2.10 d (16.0)	C: 14, 16, 32	H-8
16	214.4 C			
17	61.2 CH	2.88 s	C: 12, 13, 16, 18, 20, 21	H-12, H-23, H-32
18	176.6 C			
19	21.8 CH_3	1.27 s	C: 1, 5, 9, 10	H-1, H-2, H-8, H-30
20	83.4 C			
21	26.5 CH_3	1.47 s	C: 17, 20, 22	H-12, H-17, H-23
22	38.6 CH_2	1.80 m		
		1.59 m		
23	22.9 CH_2	2.28 m		
		2.04 m		
24	123.7 CH	5.03 m		H-22
25	132.0 C			
26	25.4 CH_3	1.55 s	C: 24, 25, 27	H-24
27	17.4 CH_3	1.52 s	C: 24, 25, 26	H-23
30	16.4 CH_3	0.96 s	C: 3, 4, 5, 31	H-2, H-6, H-19, H-31
31	27.8 CH_3	1.13 s	C: 3, 4, 5, 30	H-3, H-5, H-6, H-30
32	20.5 CH_3	0.89 s	C: 8, 13, 14, 15	H-7, H-12, H-15, H-17

[a] Recorded at 176.04 MHz in C_5D_5N. [b] Recorded at 700.13 MHz in C_5D_5N.

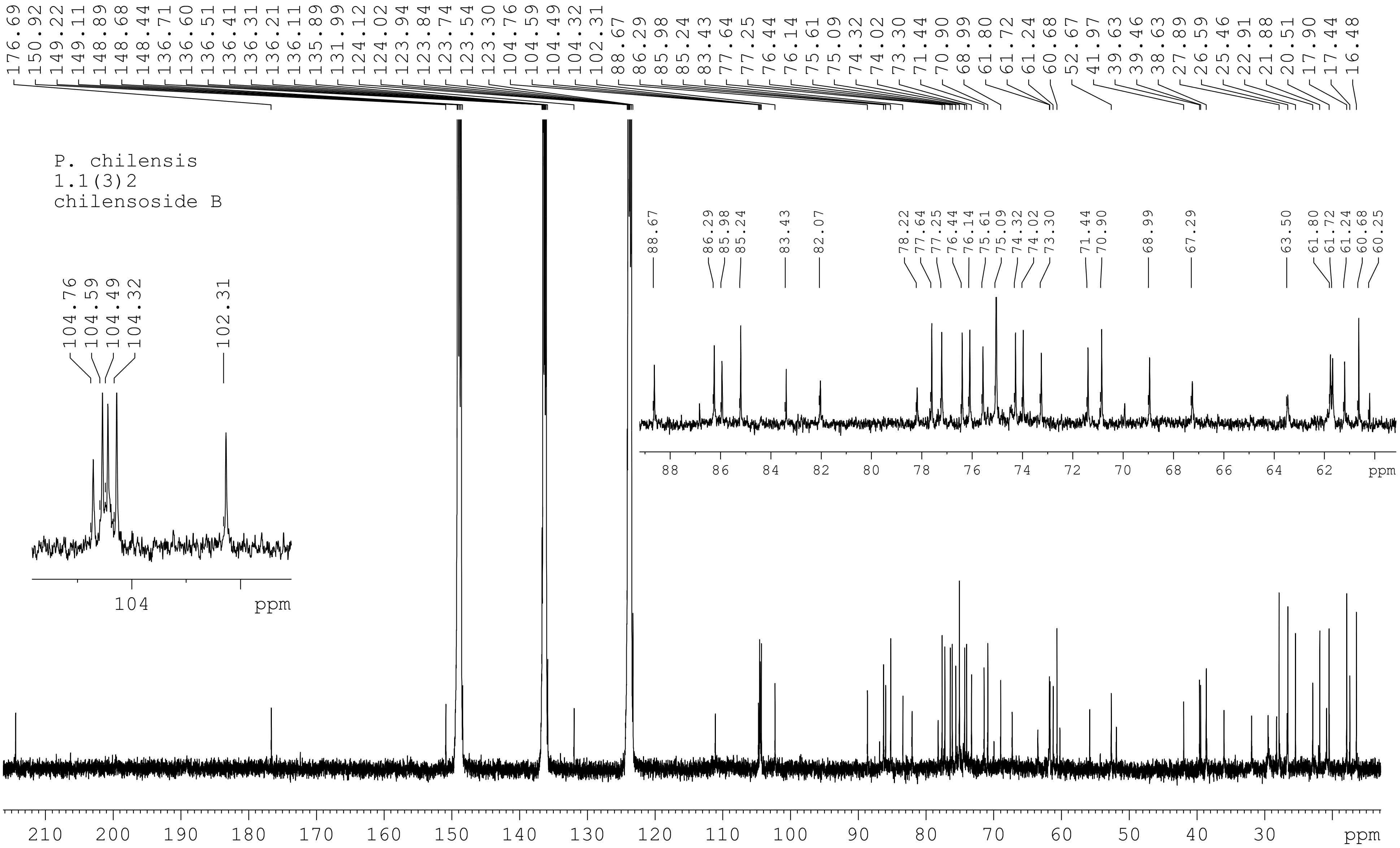

Figure S17. The ^{13}C NMR (176.04 MHz) spectrum of chilensoside B (**3**) in C_5D_5N/D_2O (4/1)

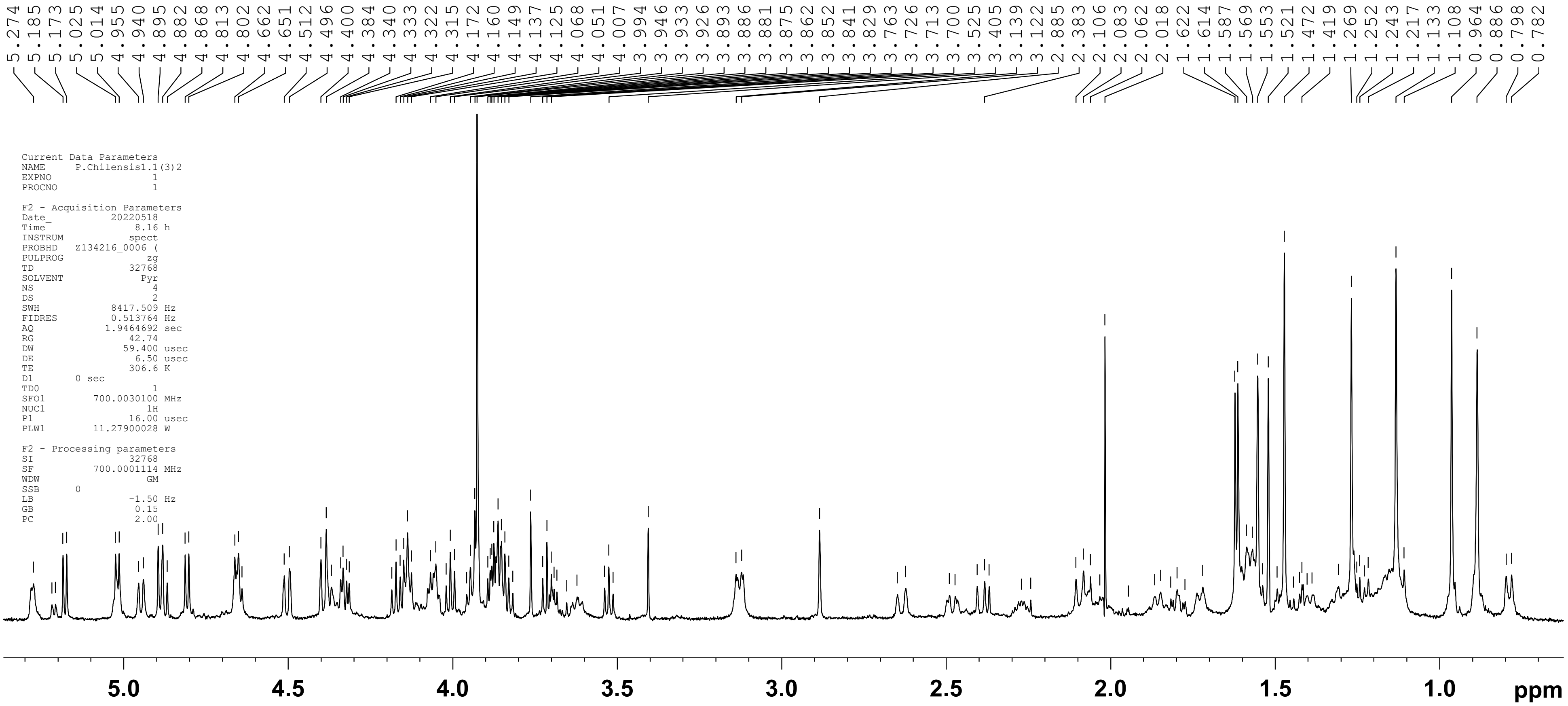

Figure S18. The ^{1}H NMR (700.13 MHz) spectrum of chilensoside B (**3**) in C_5D_5N/D_2O (4/1)

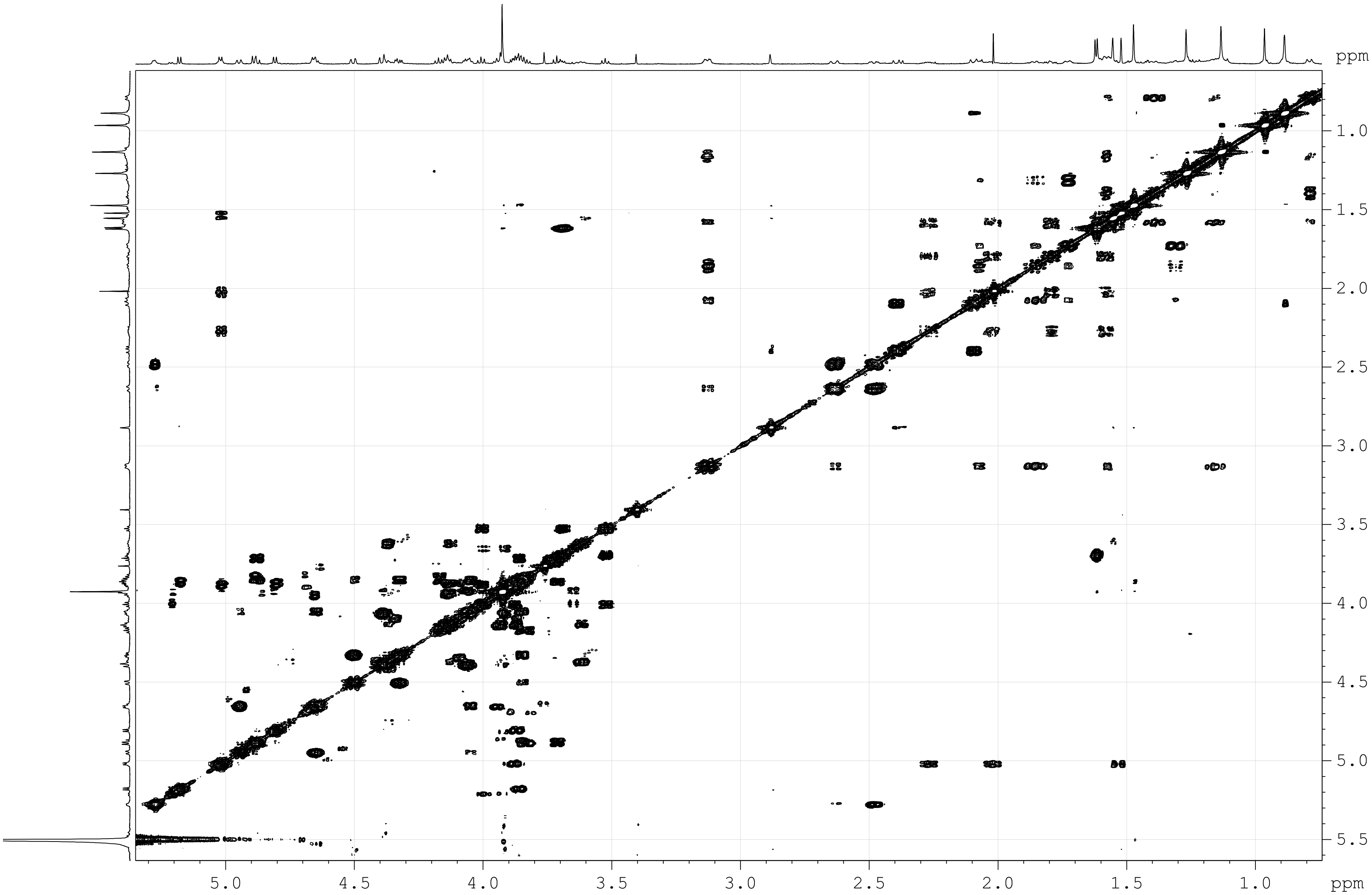

Figure S19. The COSY (700.13 MHz) spectrum of chilensoside B (**3**) in C_5D_5N/D_2O (4/1)

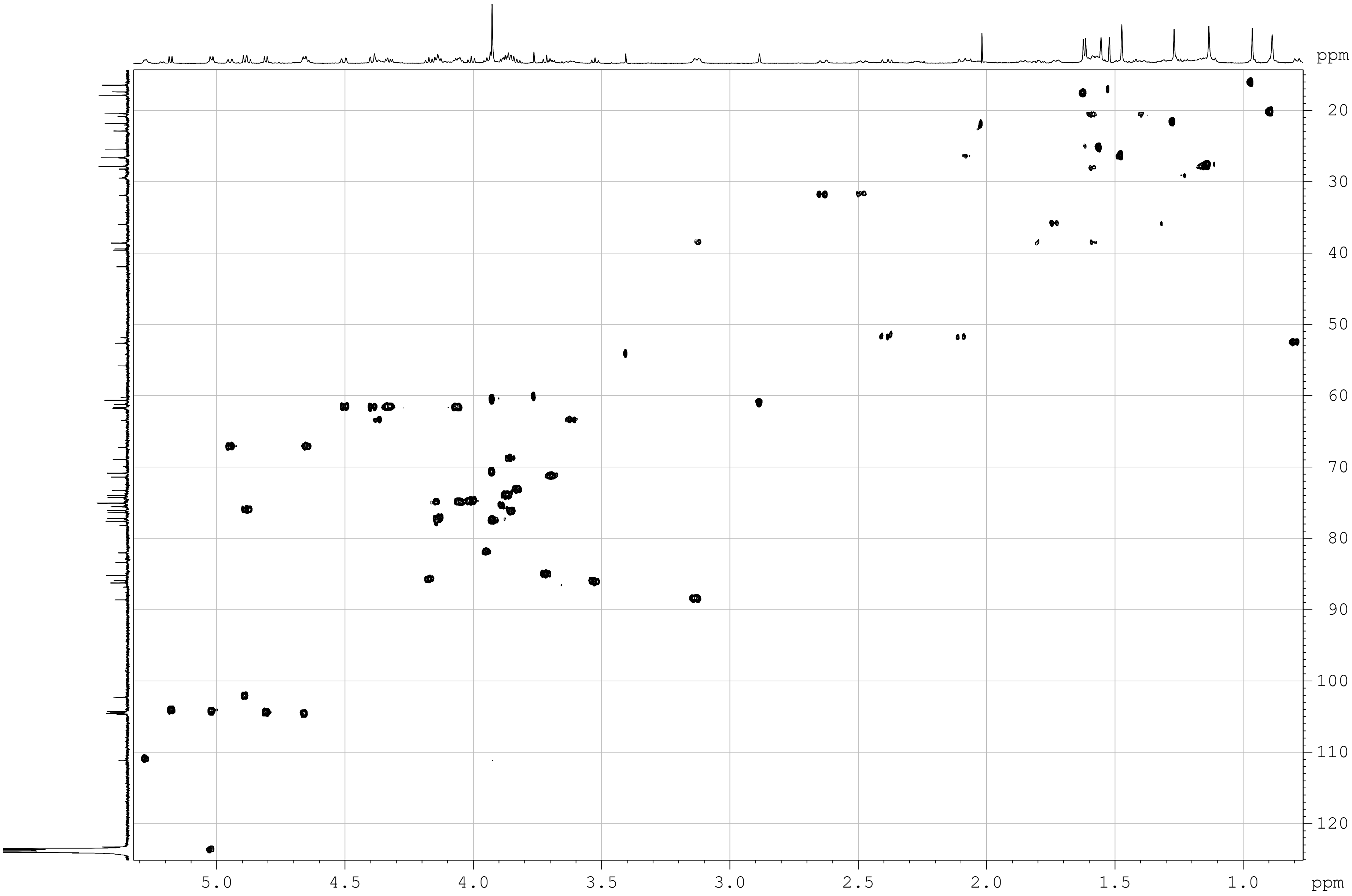

Figure S20. The HSQC (700.13 MHz) spectrum of chilensoside B (**3**) in C_5D_5N/D_2O (4/1)

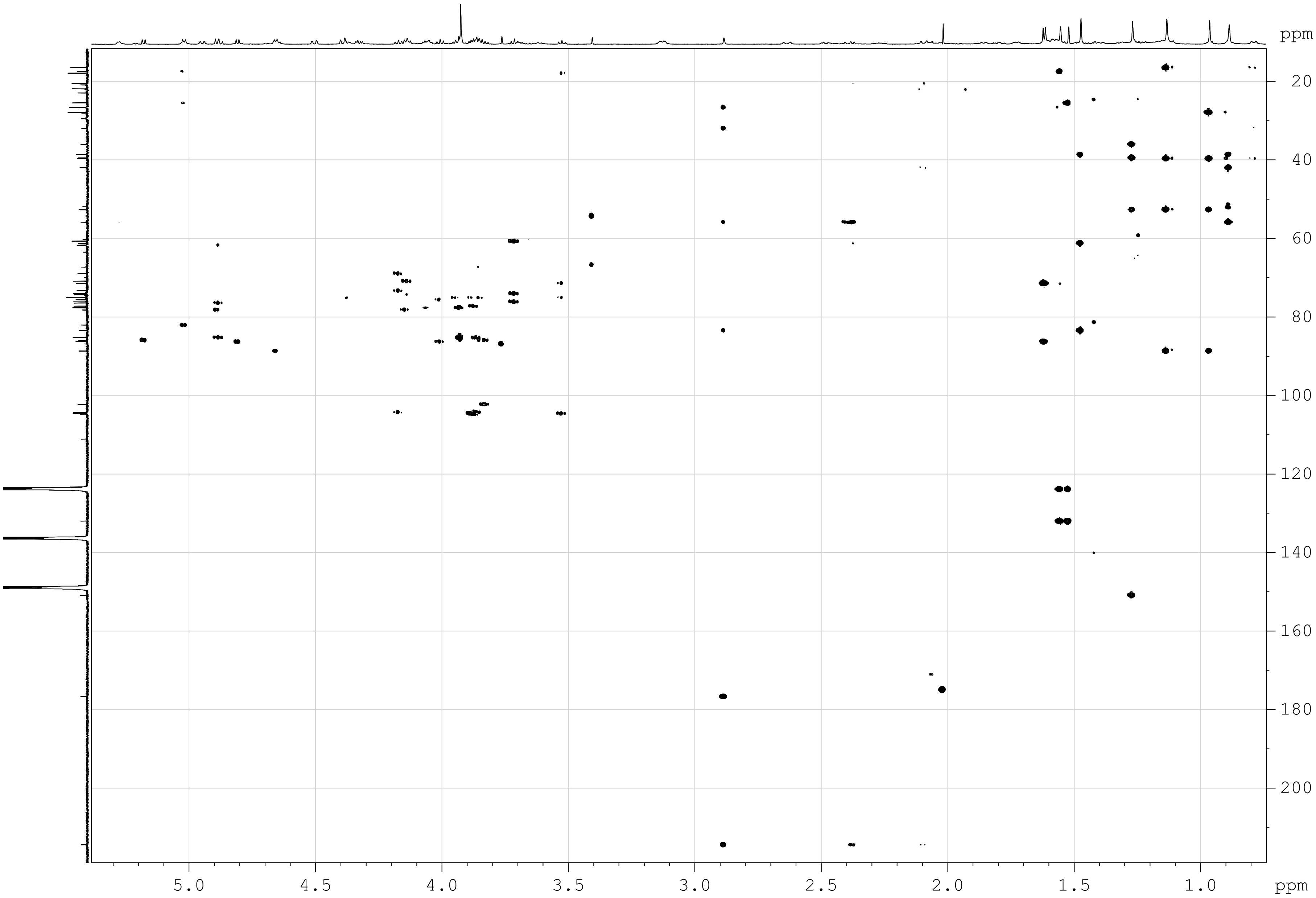

Figure S21. The HMBC (700.13 MHz) spectrum of chilensoside B (**3**) in C_5D_5N/D_2O (4/1)

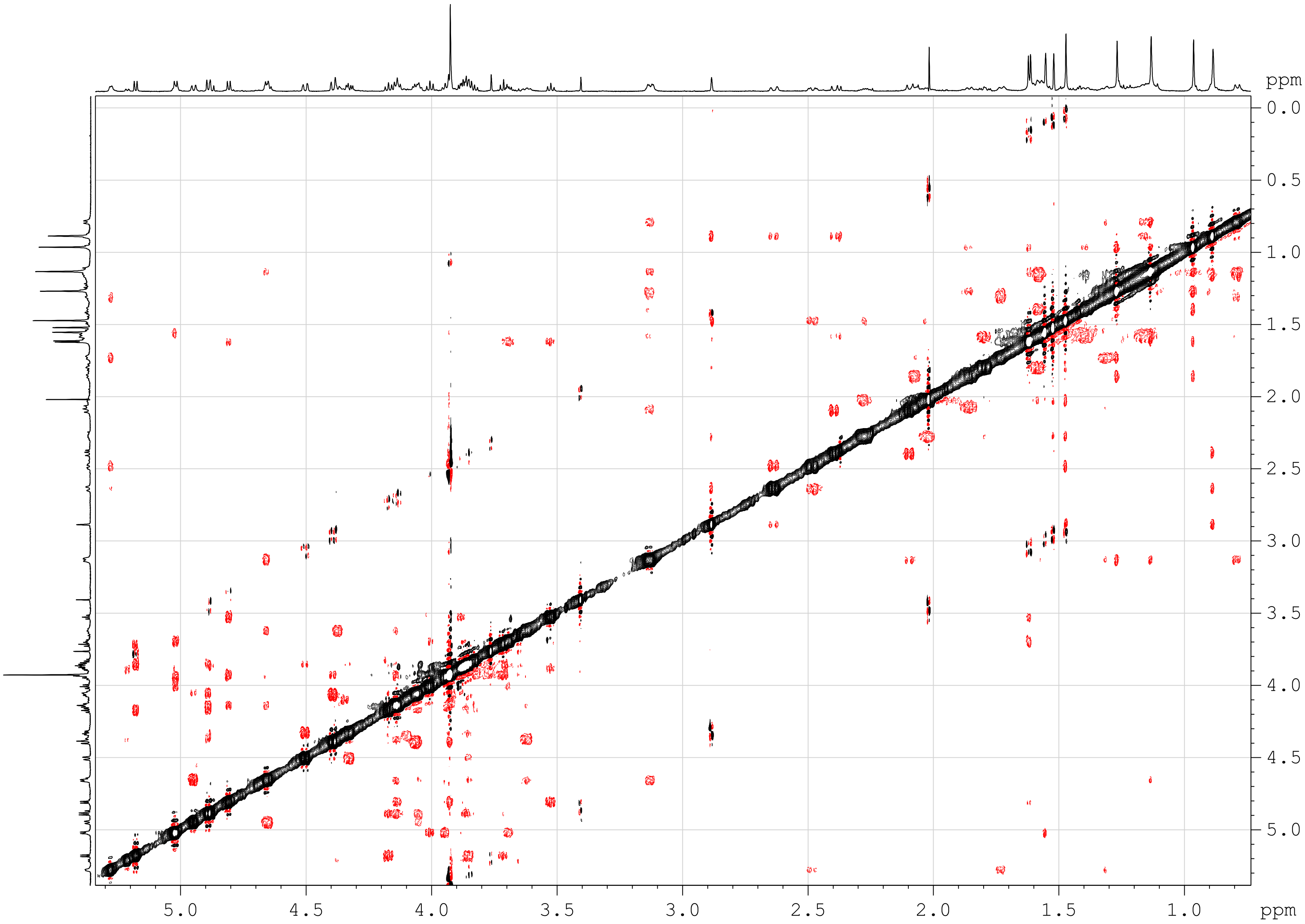

Figure S22. The ROESY (700.13 MHz) spectrum of chilensoside B (**3**) in C_5D_5N/D_2O (4/1)

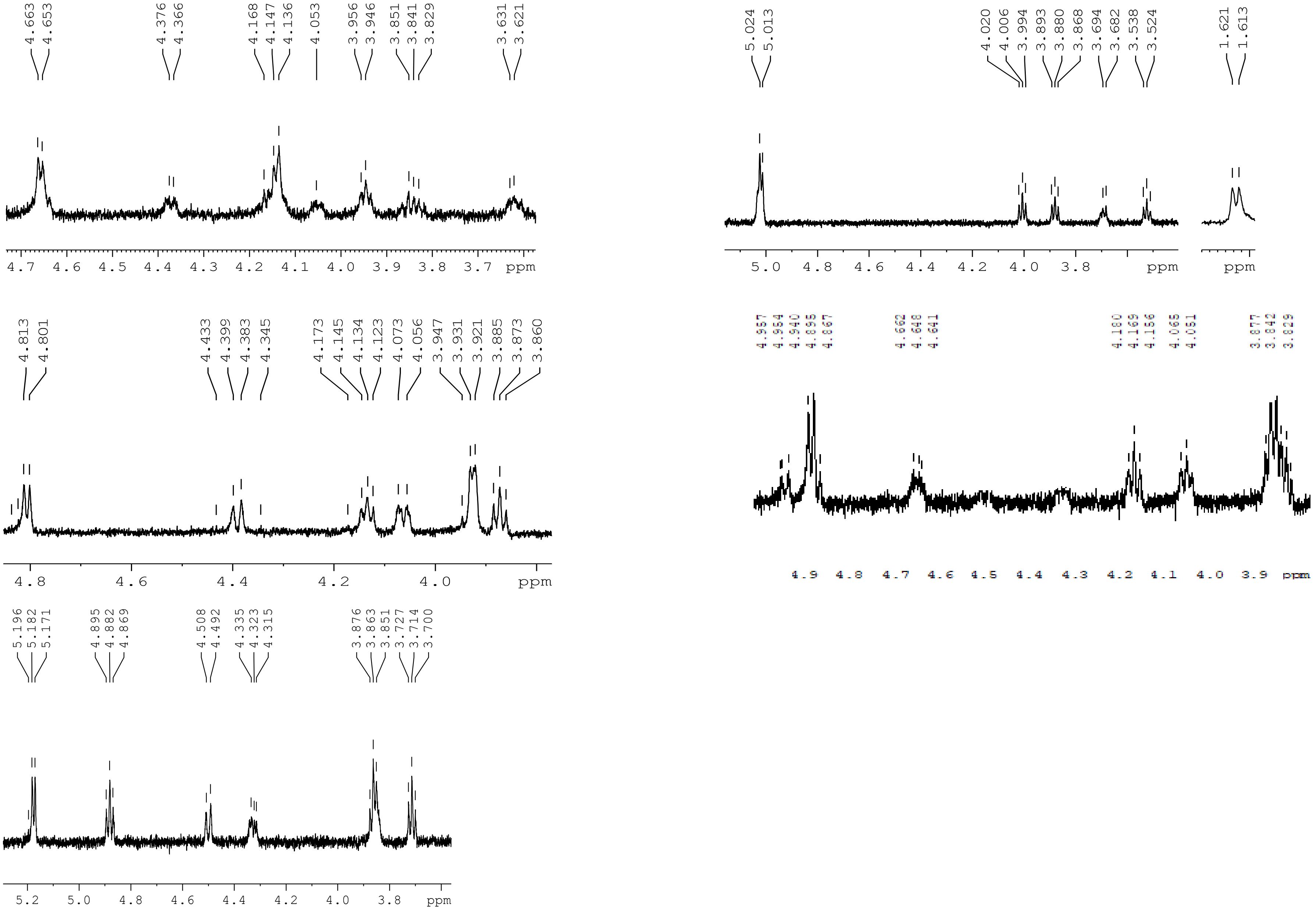

Figure S23. 1 D TOCSY (700.13 MHz) spectra of Xyl1, Qui2, Glc3, Glc4, MeGlc5 of chilensoside B (**3**) in C_5D_5N/D_2O (4/1)

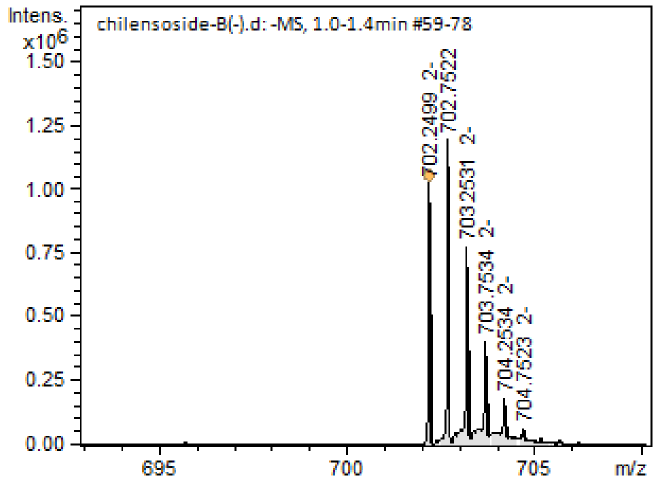

∗ The isotopic composition in the HR-ESI-MS of chilensosides A–B (**1–3**), where the ion peak of $[M_{Na}+1]^{-}$ is more intensive, than that of $[M_{Na}]^{-}$ is explained by the easy exchange of the protons at C-15, adjacent to 16-oxo-group, to deuterium during the forced long-term storage of the samples in C_5D_5N/D_2O for the registration of the NMR spectra.

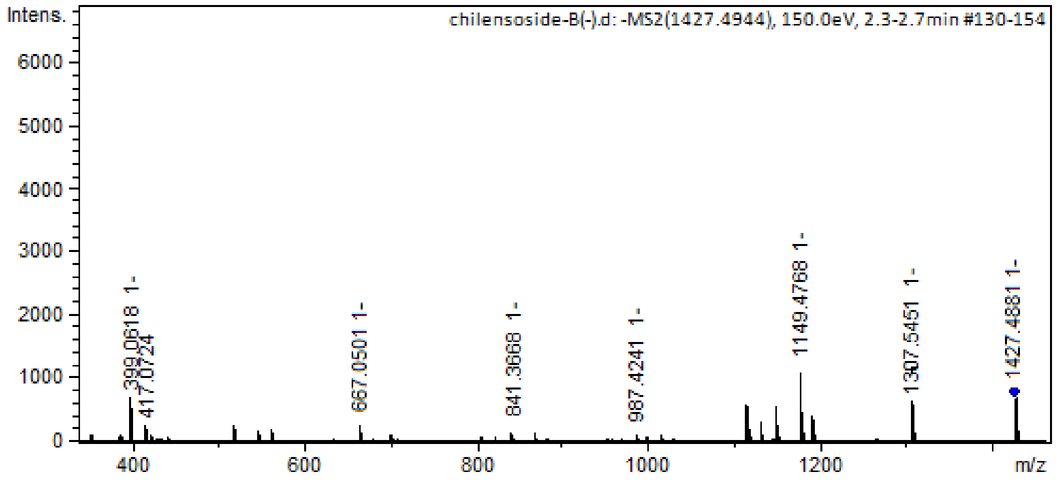

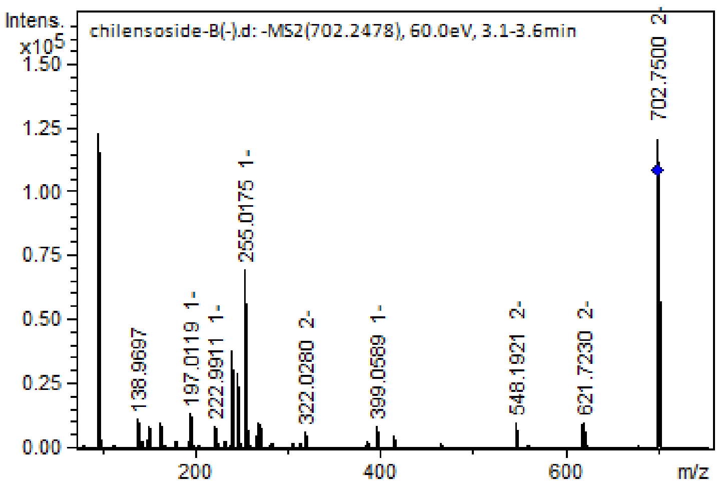

Figure S24. HR-ESI-MS and ESI-MS/MS spectra of chilensoside B (**3**)

Table S3. ^{13}C and ^{1}H NMR chemical shifts, HMBC and ROESY correlations of the aglycone moiety of chilensoside C (**4**).

Position	δ_C mult.[a]	δ_H mult. (*J* in Hz)[b]	HMBC	ROESY
1	36.0 CH_2	1.73 m		H-11
		1.31 m		H-3
2	26.7 CH_2	2.06 m		
		1.85 m		H-19, H-30
3	88.6 CH	3.13 dd (5.4; 10.9)		H-1, H-5, H-31, H1-Xyl1
4	39.4 C			
5	52.7 CH	0.79 brd (10.9)	C: 4, 6, 19, 30	H-1, H-3, H-7
6	20.9 CH_2	1.61 m		
		1.41 m		H-8, H-30
7	28.3 CH_2	1.60 m		H-15
		1.17 m		H-5, H-32
8	38.6 CH	3.13 m		H-6, H-15, H-19
9	151.0 C			
10	39.6 C			
11	111.2 CH	5.27 brd (5.8)	C: 10, 13	H-1
12	31.9 CH_2	2.63 brd (16.8)	C: 11, 18	H-17, H-32
		2.47 dd (5.8; 16.8)	C: 11, 14	H-17, H-21
13	55.8 C			
14	42.0 C			
15	51.9 CH_2	2.40 d (15.4)	C: 13, 16, 17, 32	
		2.11 d (15.4)	C: 14, 16, 32	H-8
16	214.4 C			
17	61.2 CH	2.87 s	C: 12, 13, 16, 18, 20, 21	H-12, H-23, H-32
18	176.7 C			
19	21.9 CH_3	1.28 s	C: 1, 5, 9, 10	H-1, H-2, H-8, H-30
20	83.4 C			
21	26.6 CH_3	1.47 s	C: 17, 20, 22	H-12, H-17, H-23
22	38.6 CH_2	1.80 m		
		1.59 m		
23	22.9 CH_2	2.28 m		H-21
		2.04 m		
24	123.8 CH	5.03 m		H-22
25	132.0 C			
26	25.5 CH_3	1.55 s	C: 24, 25, 27	H-24
27	17.4 CH_3	1.52 s	C: 24, 25, 26	H-23
30	16.4 CH_3	0.95 s	C: 3, 4, 5, 31	H-2, H-6, H-19, H-31
31	27.9 CH_3	1.13 s	C: 3, 4, 5, 30	H-3, H-5, H-6, H-30
32	20.5 CH_3	0.88 s	C: 8, 13, 14, 15	H-7, H-12, H-15, H-17

[a] Recorded at 176.04 MHz in C_5D_5N. [b] Recorded at 700.13 MHz in C_5D_5N.

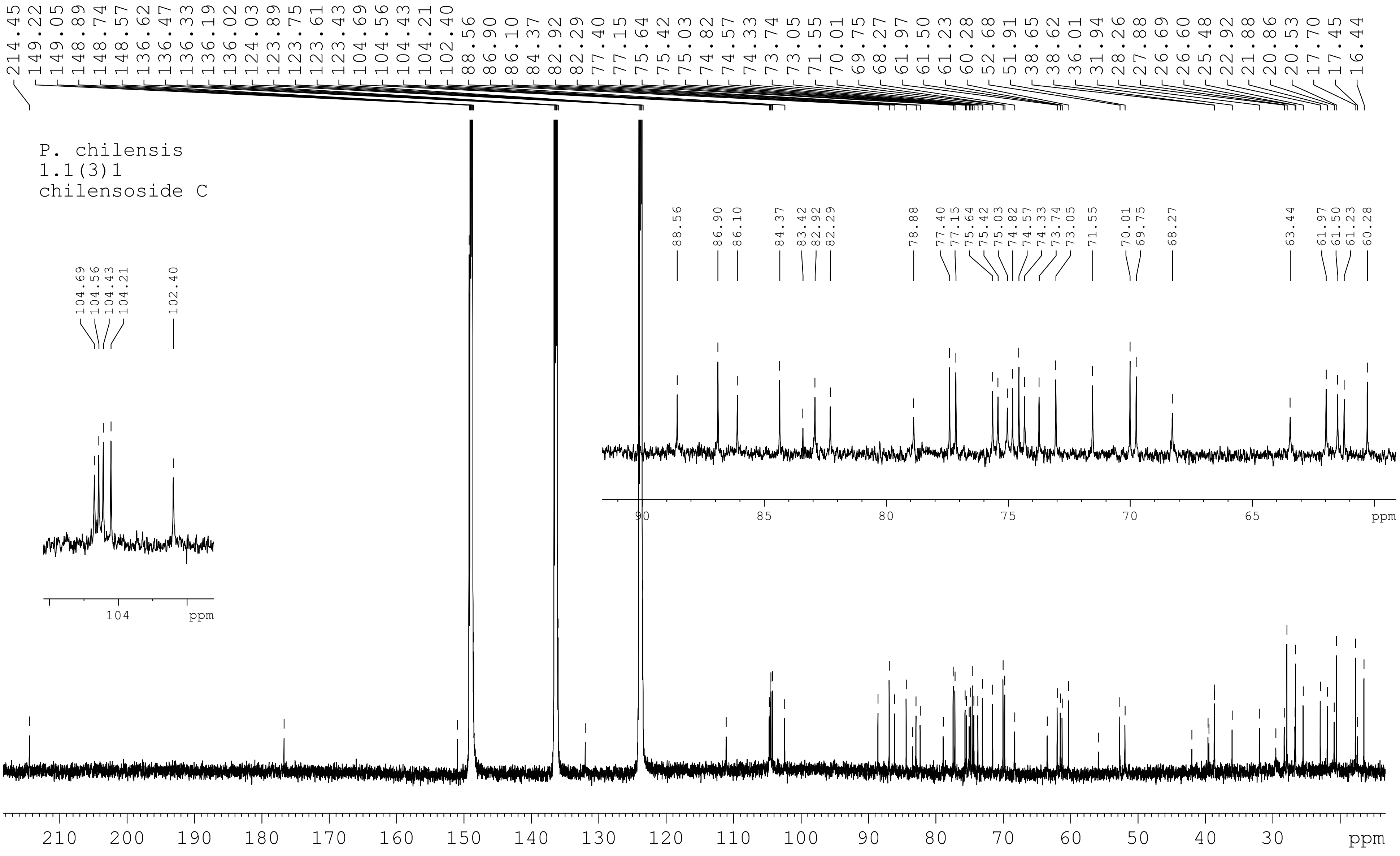

Figure S25. The ^{13}C NMR (176.04 MHz) spectrum of chilensoside C (**4**) in C_5D_5N/D_2O (4/1)

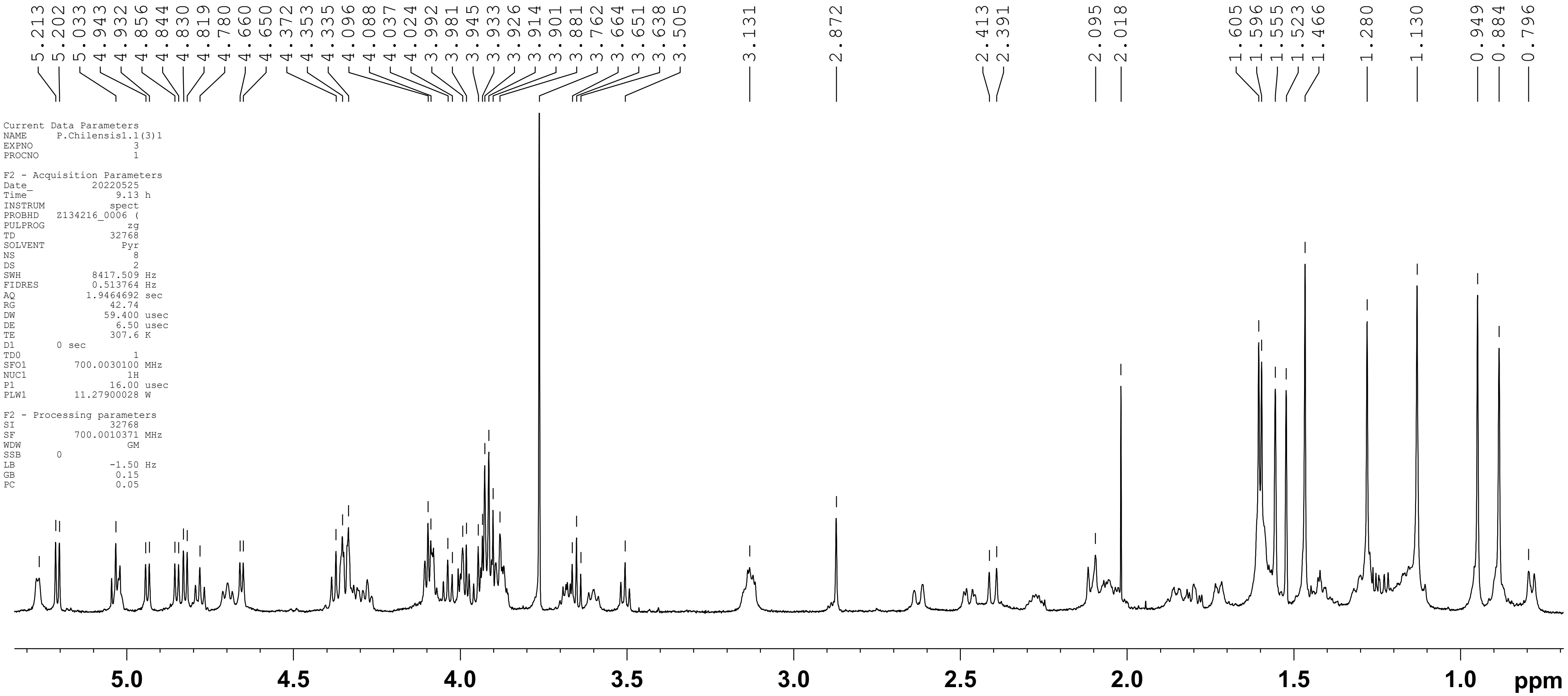

Figure S26. The ^{1}H NMR (700.13 MHz) spectrum of chilensoside C (**4**) in C_5D_5N/D_2O (4/1)

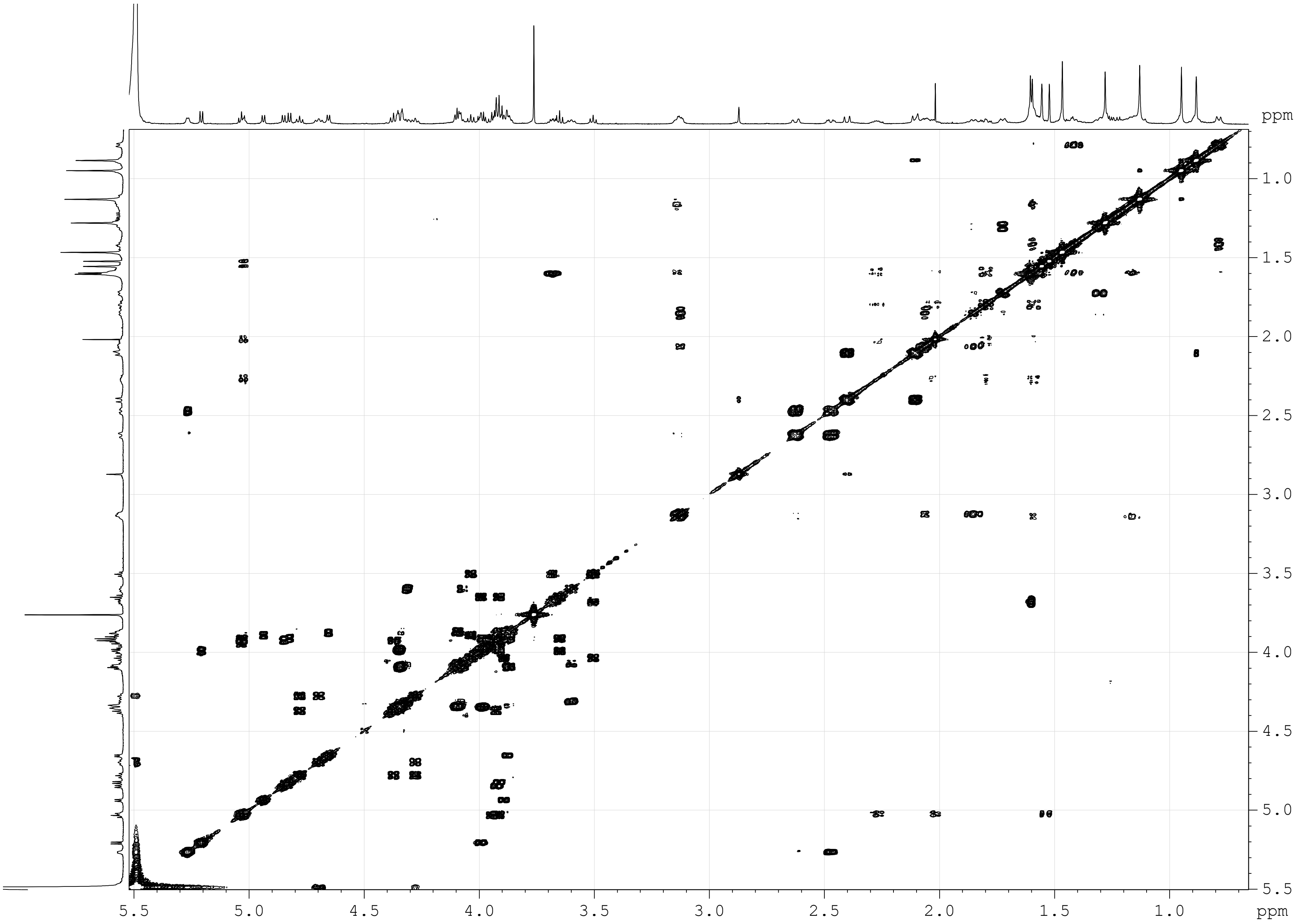

Figure S27. The COSY (700.13 MHz) spectrum of chilensoside C (**4**) in C_5D_5N/D_2O (4/1)

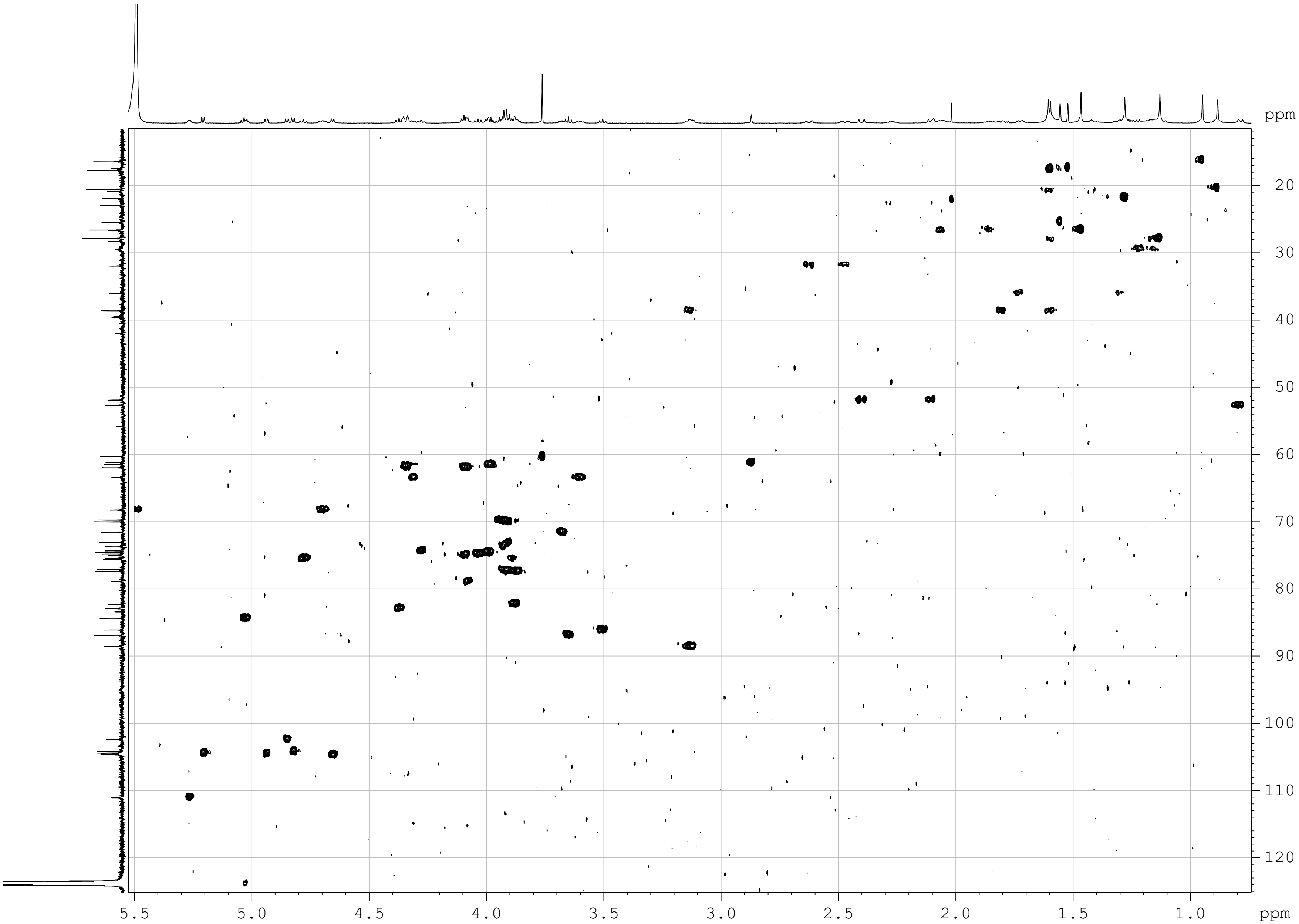

Figure S28. The HSQC (700.13 MHz) spectrum of chilensoside C (**4**) in C_5D_5N/D_2O (4/1)

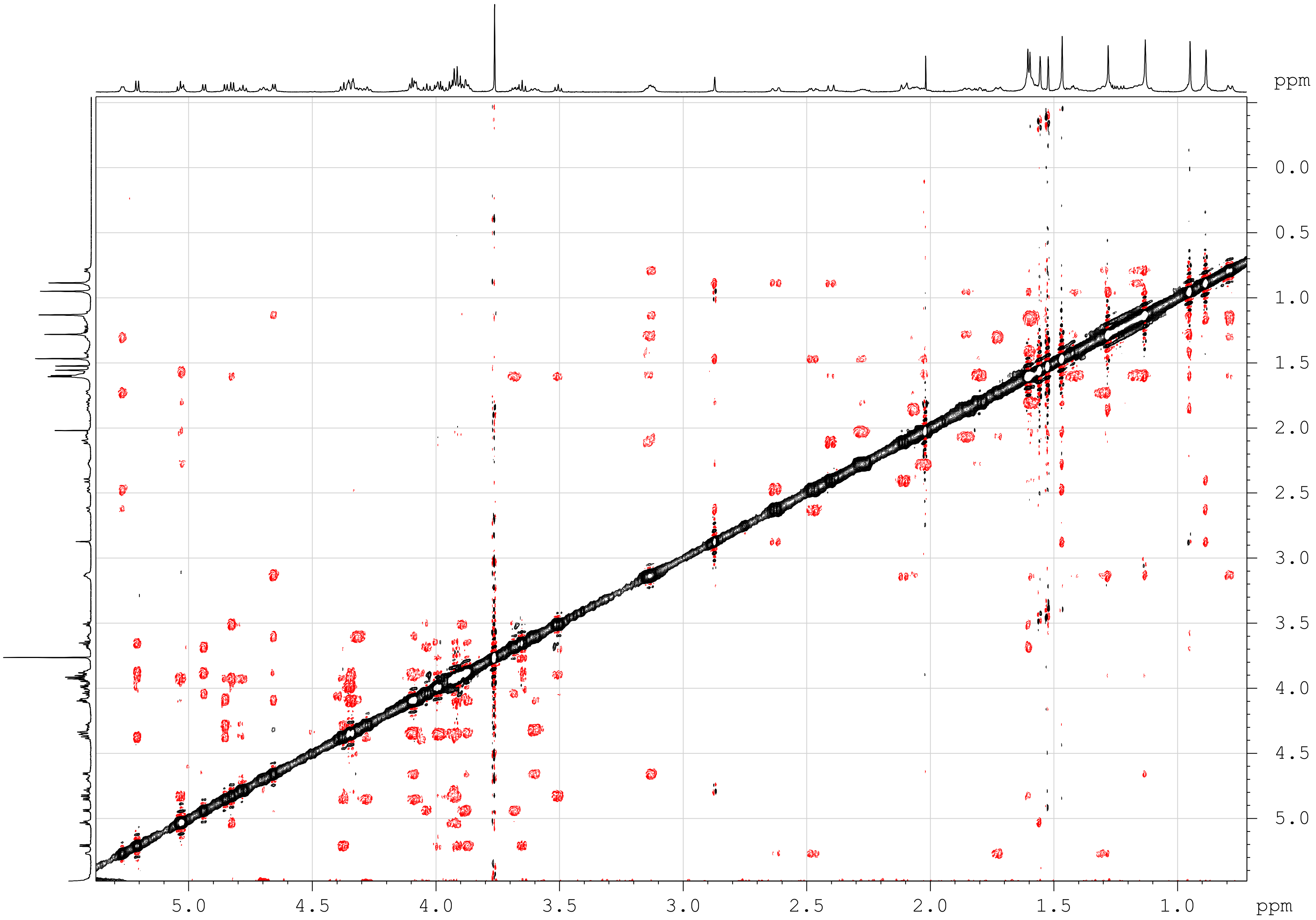

Figure S29. The ROESY (700.13 MHz) spectrum of chilensoside C (**4**) in C_5D_5N/D_2O (4/1)

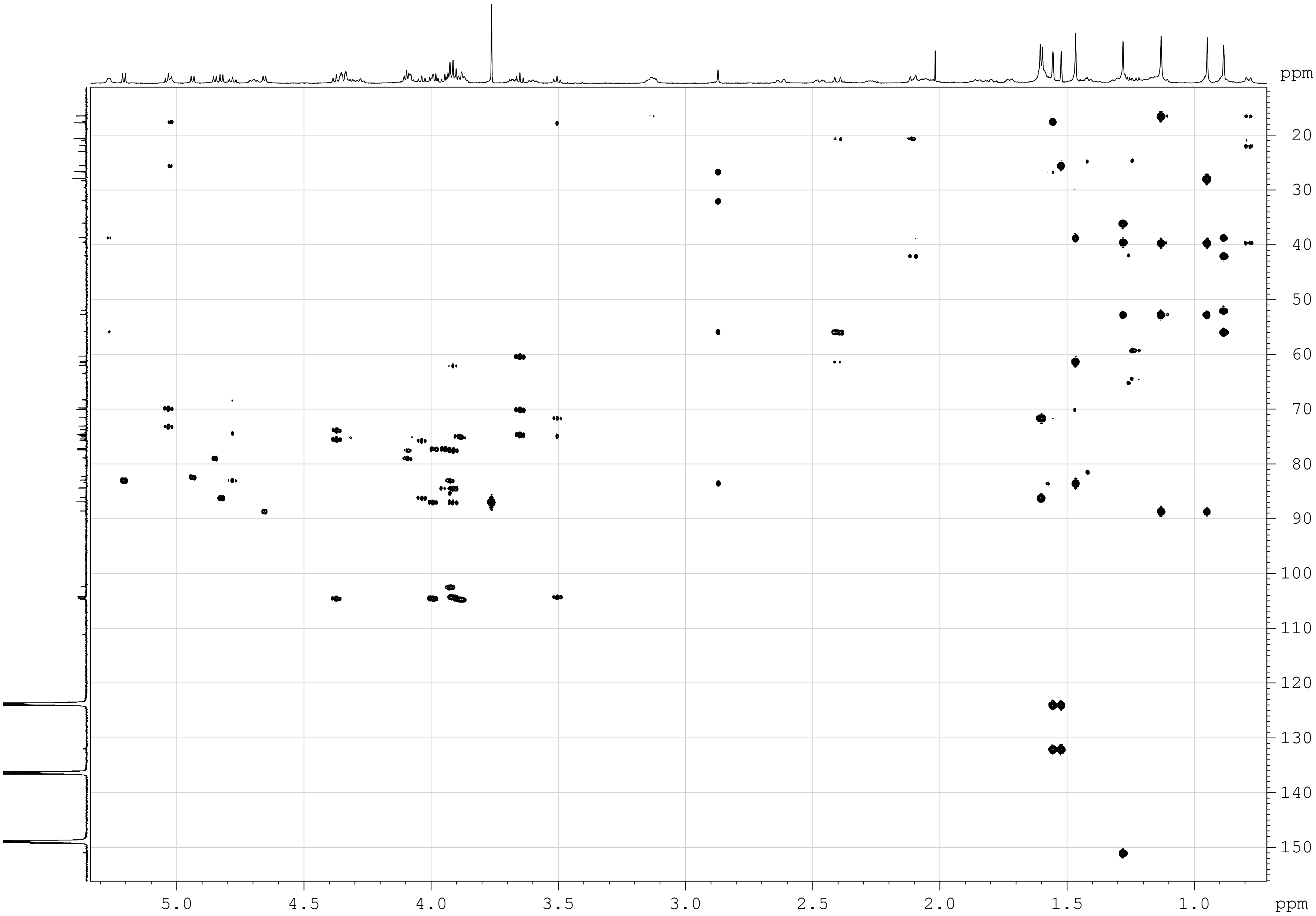

Figure S30. The HMBC (500.12 MHz) spectrum of chilensoside C (**4**) in C_5D_5N/D_2O (4/1)

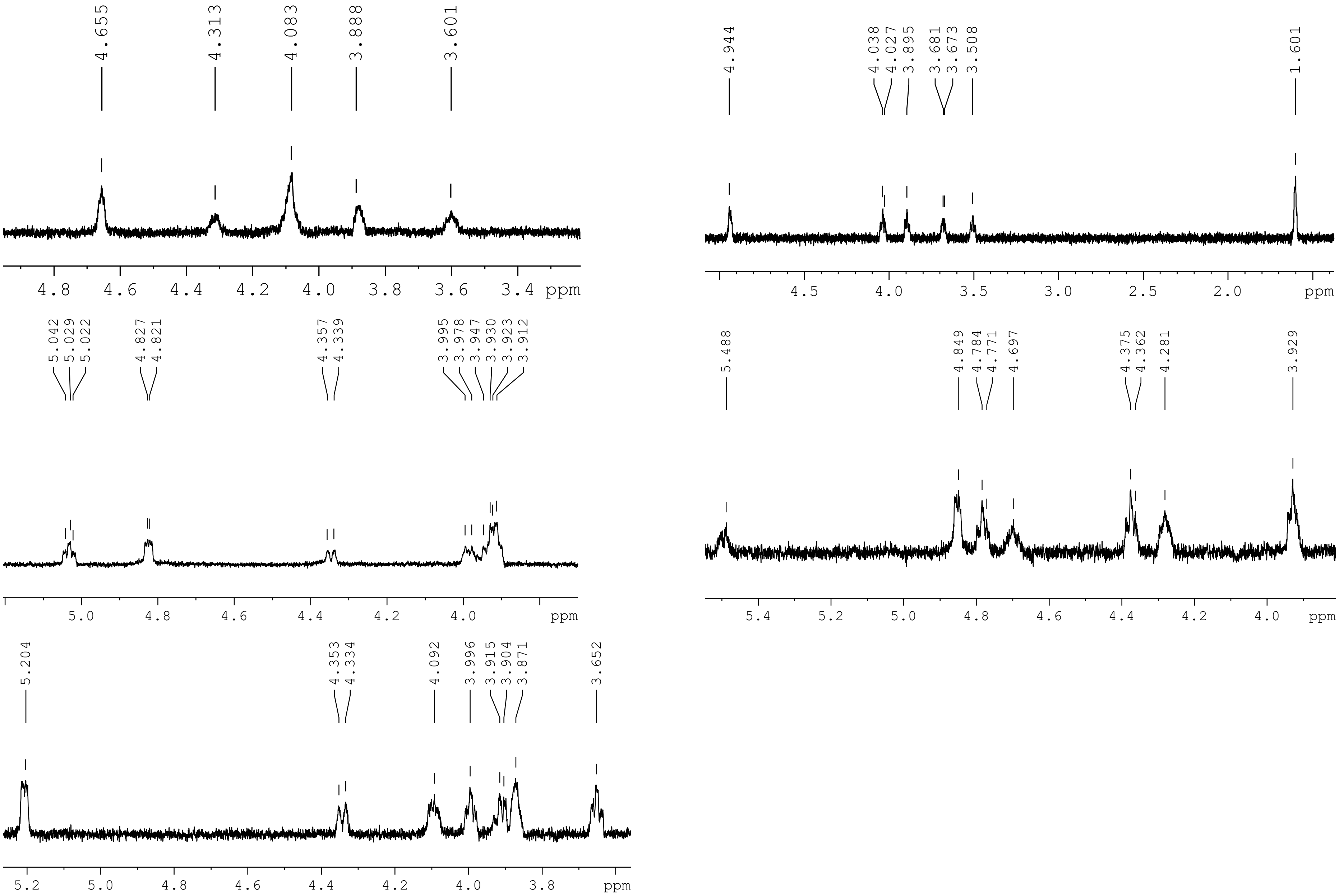

Figure S31. 1D TOCSY (700.13 MHz) spectra of Xyl1, Qui2, Glc3, Glc4, MeGlc5 of chilensosideC (**4**) in C_5D_5N/D_2O (4/1)

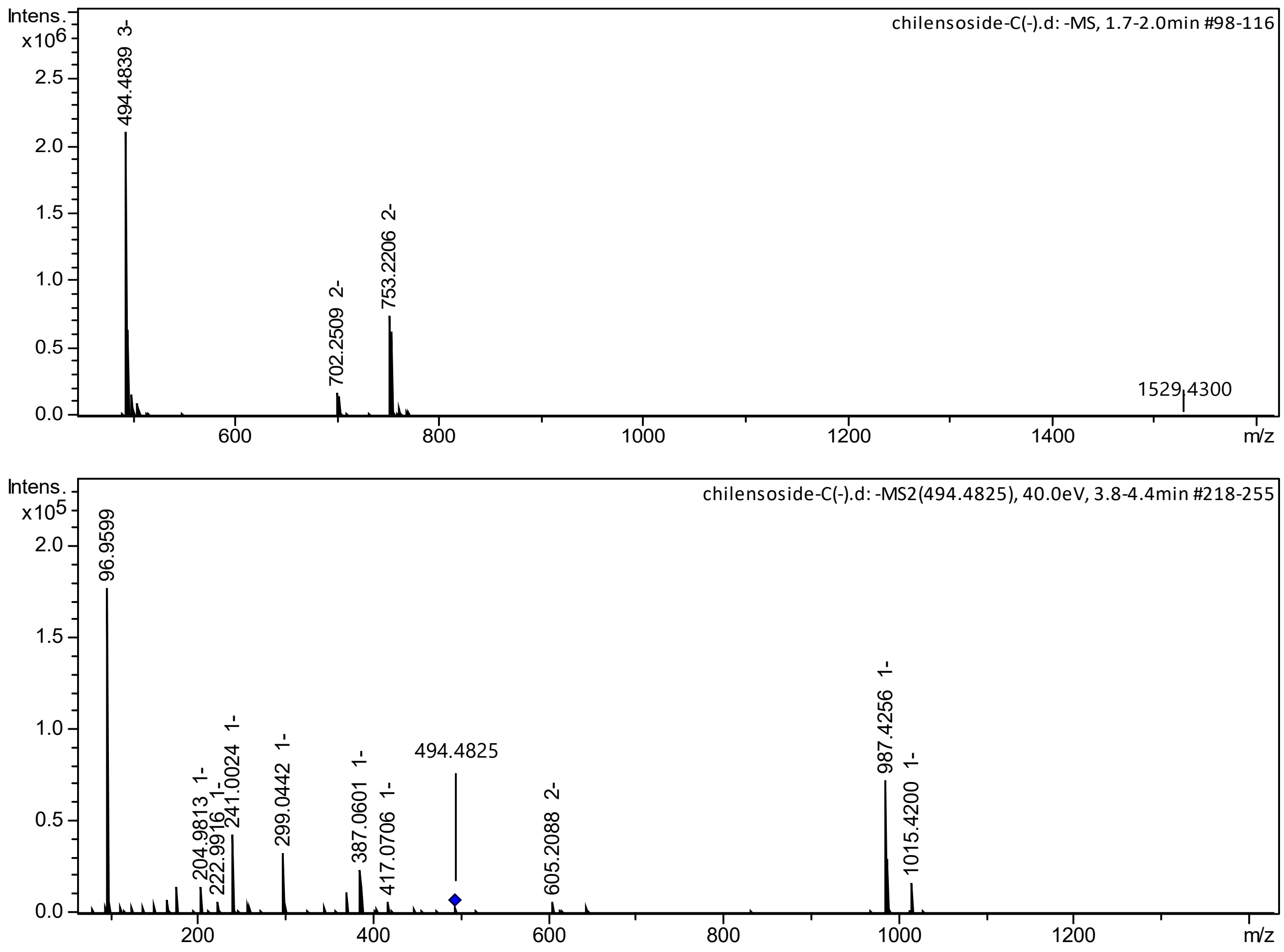

Figure S32. HR-ESI-MS and ESI-MS/MS spectra of chilensosideC (**4**)

Table S4. ^{13}C and ^{1}H NMR chemical shifts, HMBC and ROESY correlations of the aglycone moiety of chilensoside D (**5**).

Position	δ_C mult.[a]	δ_H mult. (*J* in Hz)[b]	HMBC	ROESY
1	36.0 CH_2	1.73 m		H-11
		1.32 m		H-3
2	26.7 CH_2	2.06 m		
		1.83 m		H-19, H-30
3	88.3 CH	3.11 dd (4.4; 11.5)		H-1, H-5, H-31, H1-Xyl1
4	39.4 C			
5	52.5 CH	0.78 brd (11.5)	C: 4, 6, 19, 30	H-1, H-3, H-7
6	20.9 CH_2	1.59 m		
		1.40 m		H-8, H-30
7	28.3 CH_2	1.60 m		H-15
		1.17 m		H-5, H-32
8	38.7 CH	3.13 m		H-6, H-15, H-19
9	150.8 C			
10	39.4 C			
11	111.2 CH	5.29 brd (5.2)	C: 10, 13	H-1
12	31.9 CH_2	2.65 brd (17.1)	C: 11, 18	H-17, H-32
		2.49 dd (6.0; 17.1)	C: 11, 14	H-17, H-21
13	55.8 C			
14	42.0 C			
15	51.9 CH_2	2.41 d (15.6)	C: 13, 16, 17, 32	
		2.10 d (15.6)	C: 14, 16, 32	H-8
16	214.6 C			
17	61.2 CH	2.90 s	C: 12, 13, 16, 18, 20, 21	H-12, H-23, H-32
18	176.7 C			
19	21.9 CH_3	1.29 s	C: 1, 5, 9, 10	H-1, H-2, H-8, H-30
20	83.5 C			
21	26.6 CH_3	1.48 s	C: 17, 20, 22	H-12, H-17, H-23
22	38.6 CH_2	1.80 m		
		1.60 m		
23	22.9 CH_2	2.29 m		H-21
		2.04 m		
24	124.0 CH	5.03 m		H-22
25	132.1 C			
26	25.5 CH_3	1.55 s	C: 24, 25, 27	H-24
27	17.4 CH_3	1.52 s	C: 24, 25, 26	H-23
30	16.4 CH_3	0.90 s	C: 3, 4, 5, 31	H-2, H-6, H-19, H-31
31	27.9 CH_3	1.10 s	C: 3, 4, 5, 30	H-3, H-5, H-6, H-30
32	20.5 CH_3	0.89 s	C: 8, 13, 14, 15	H-7, H-12, H-15, H-17

[a] Recorded at 125.67 MHz in C_5D_5N. [b] Recorded at 500.12 MHz in C_5D_5N.

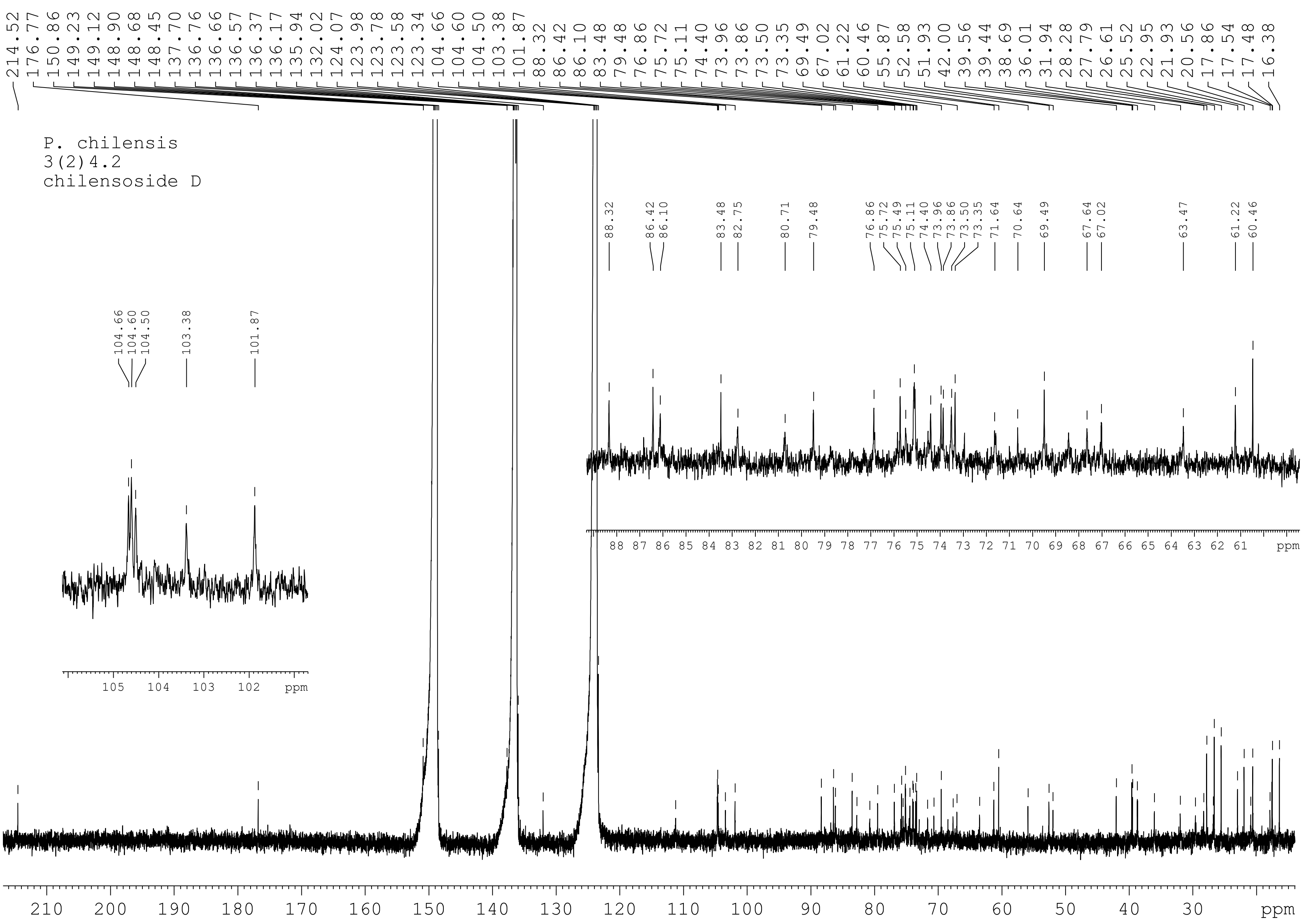

Figure S33. The ^{13}C NMR (125.67 MHz) spectrum of chilensoside D (**5**) in C_5D_5N/D_2O (4/1)

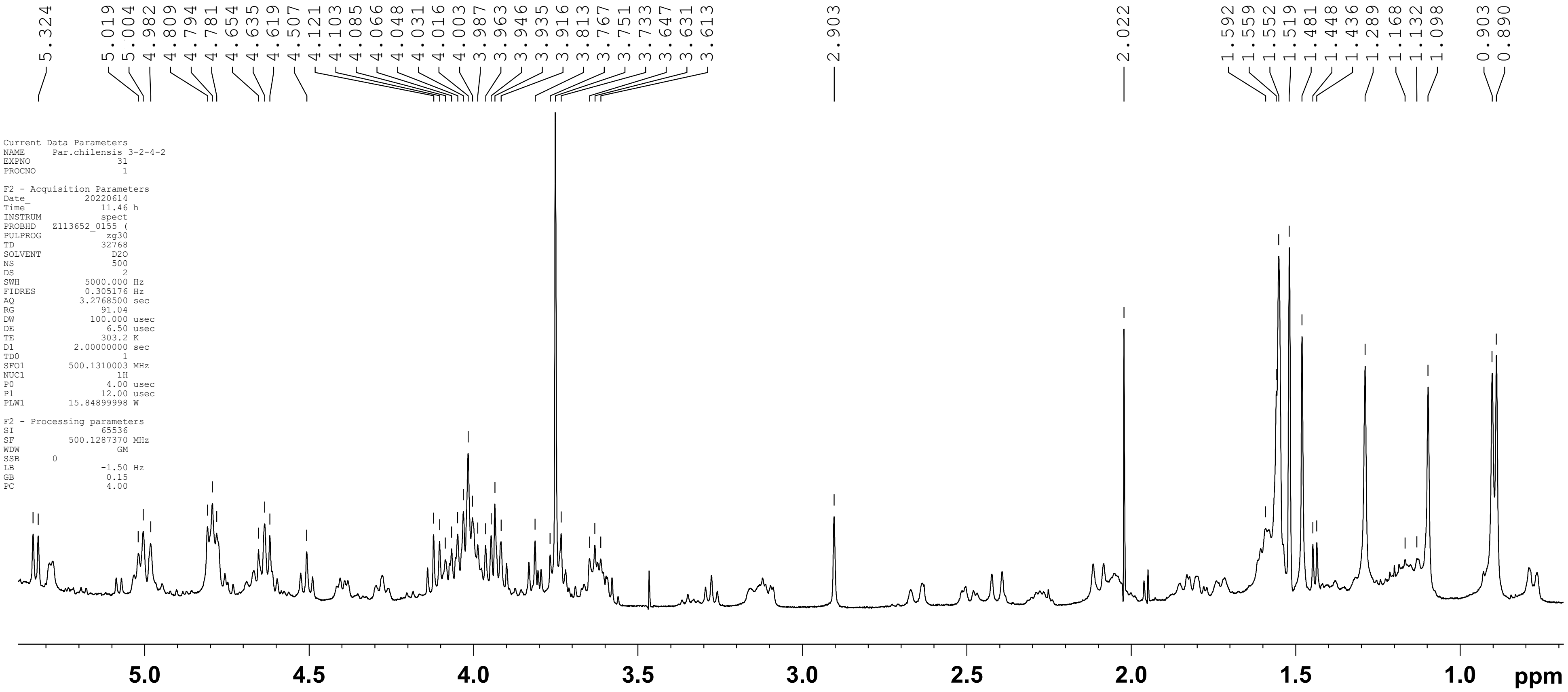

Figure S34. The ^1H NMR (500.12 MHz) spectrum of chilensoside D (**5**) in C_5D_5N/D_2O (4/1)

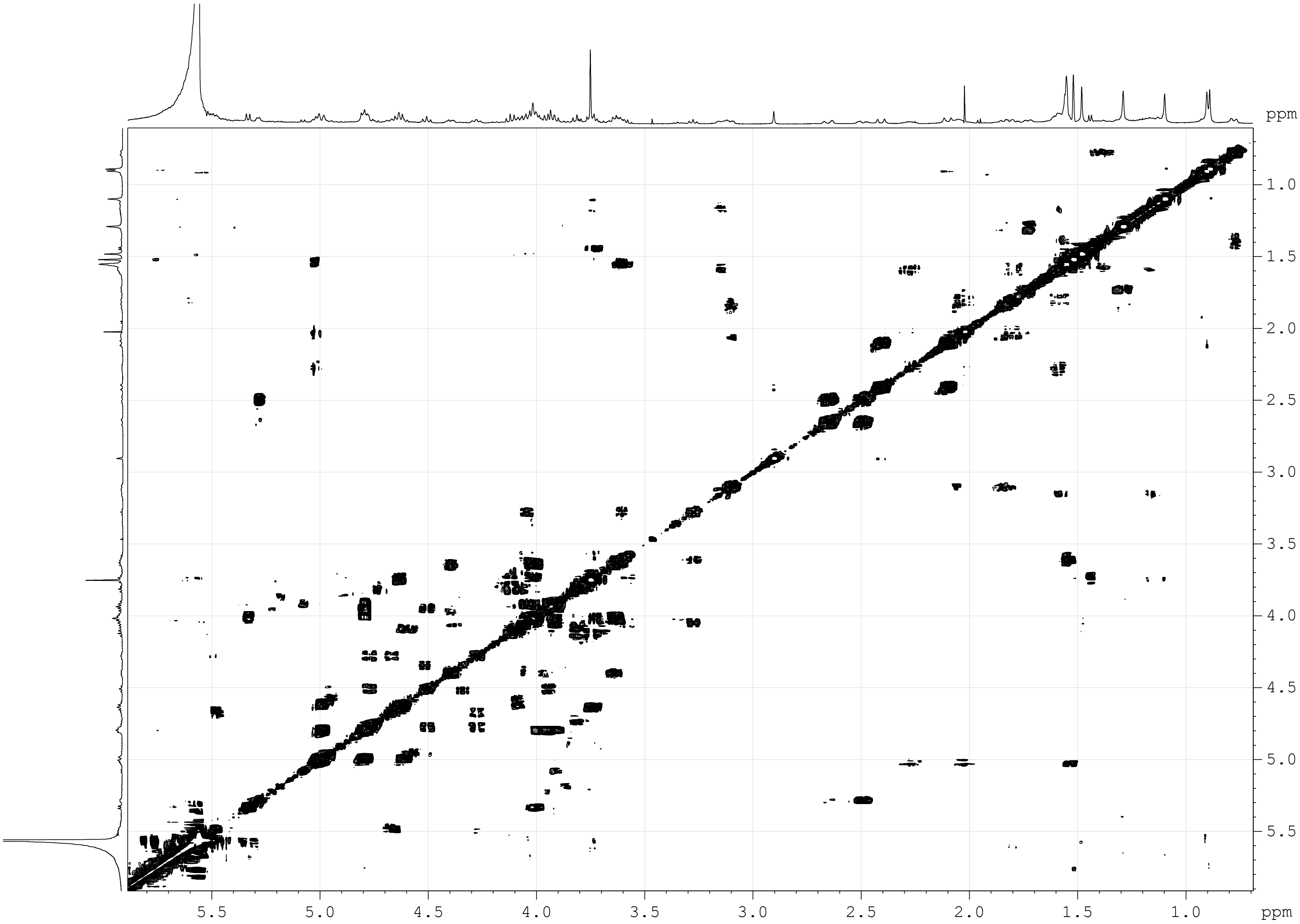

Figure S35. The COSY (500.12 MHz) spectrum of chilensoside D (**5**) in C_5D_5N/D_2O (4/1)

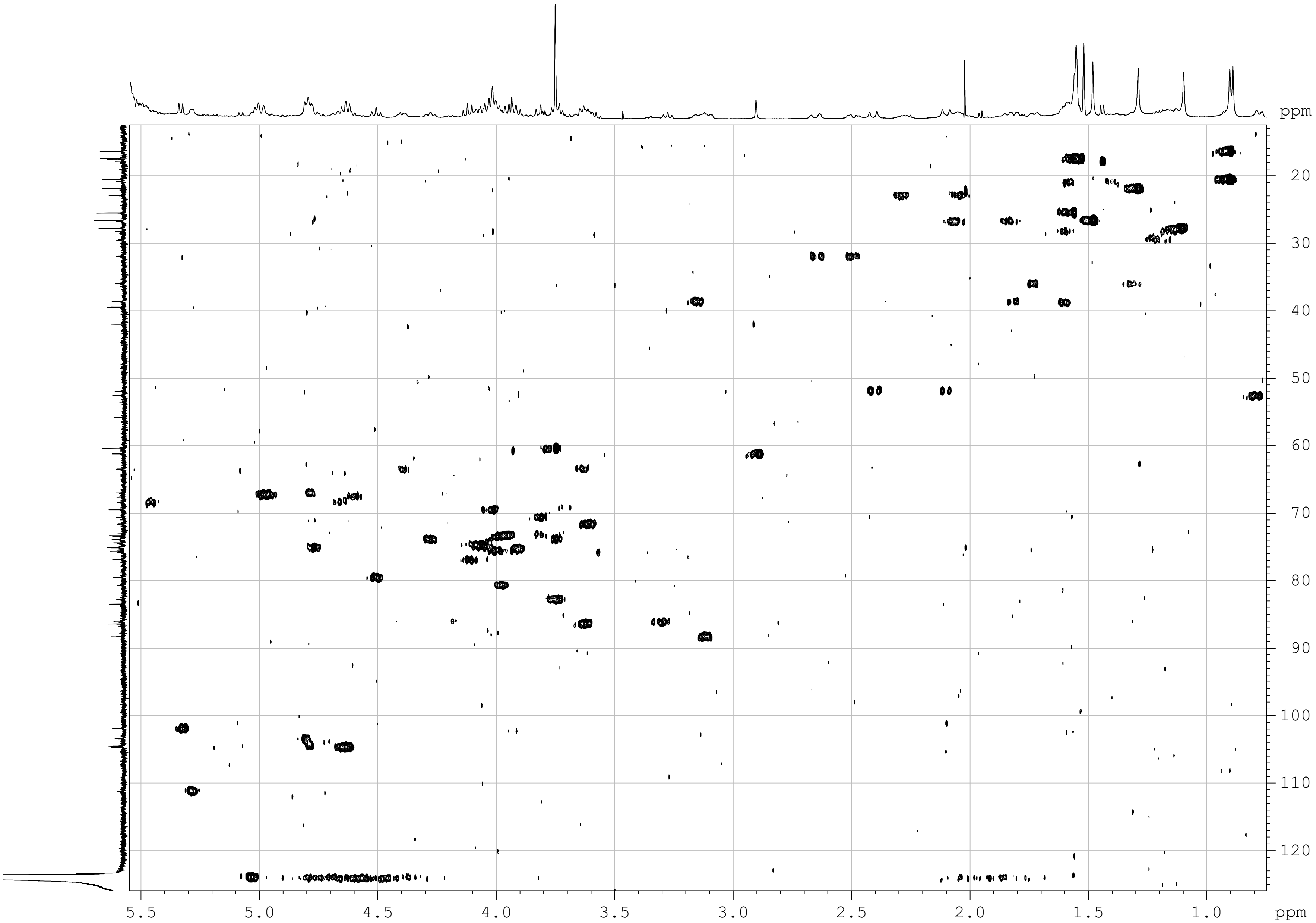

Figure S36. The HSQC (500.12 MHz) spectrum of chilensoside D (**5**) in C_5D_5N/D_2O (4/1)

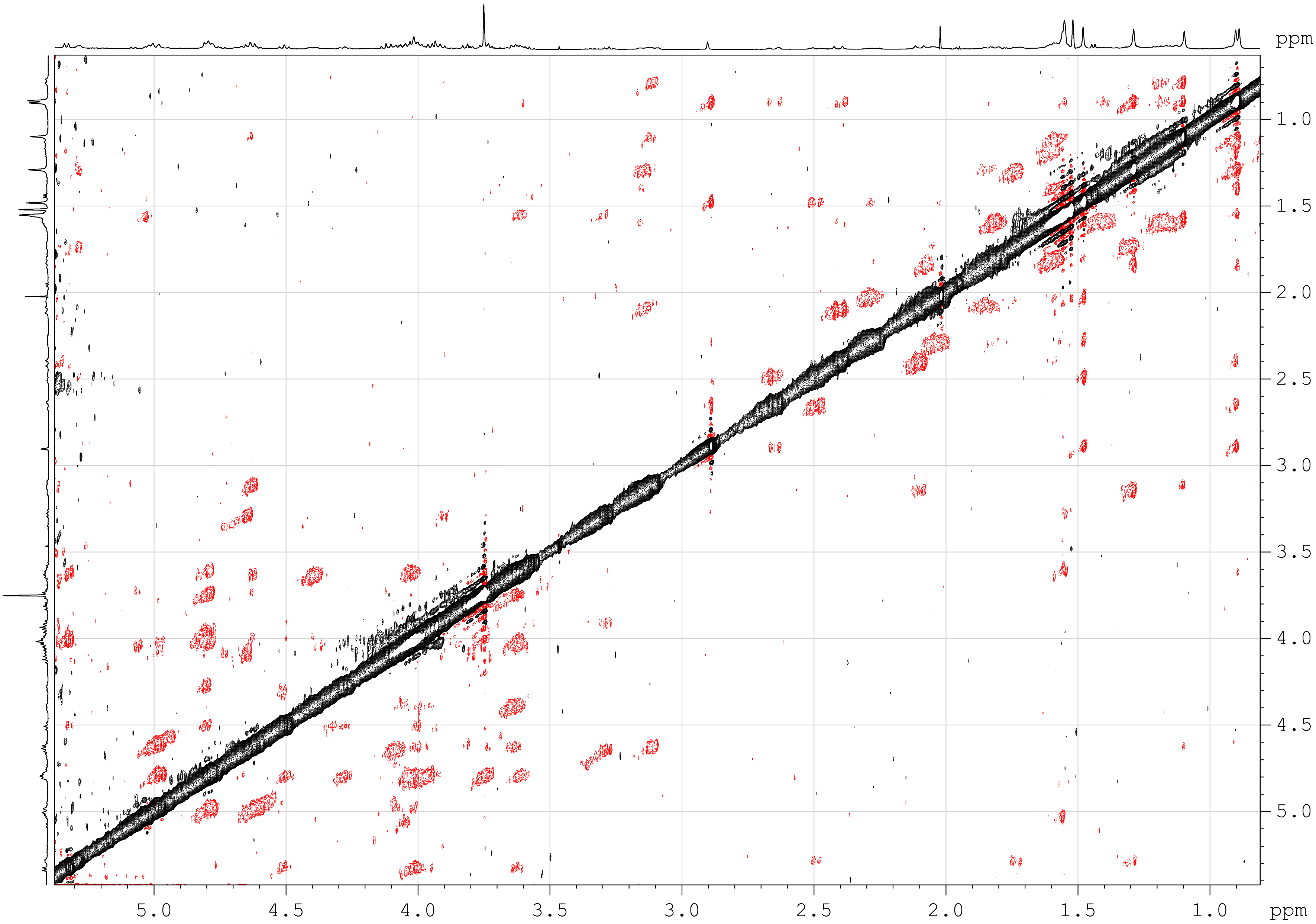

Figure S37. The ROESY (500.12 MHz) spectrum of chilensoside D (**5**) in C_5D_5N/D_2O (4/1)

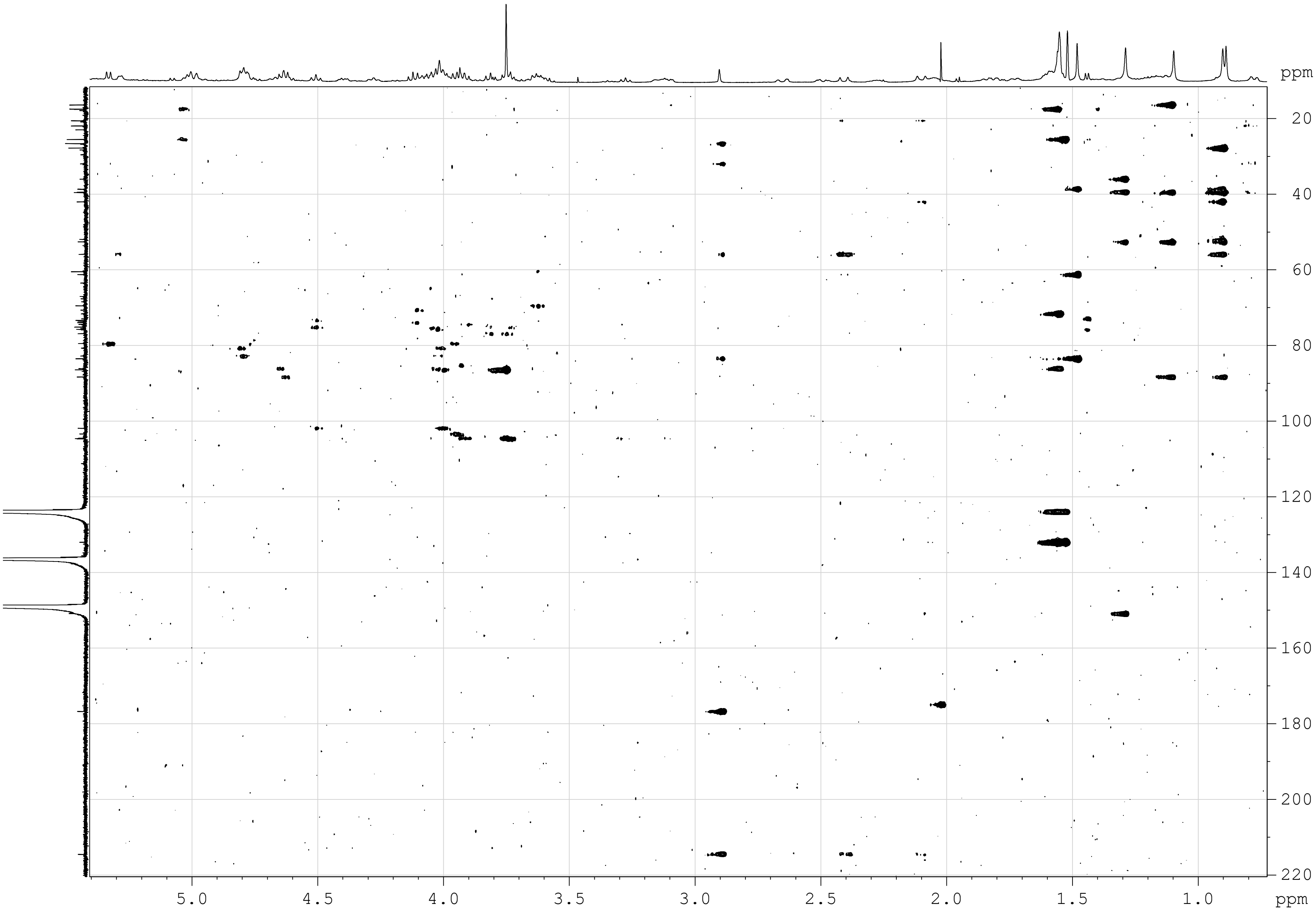

Figure S38. The HMBC (500.12 MHz) spectrum of chilensoside D (**5**) in C_5D_5N/D_2O (4/1)

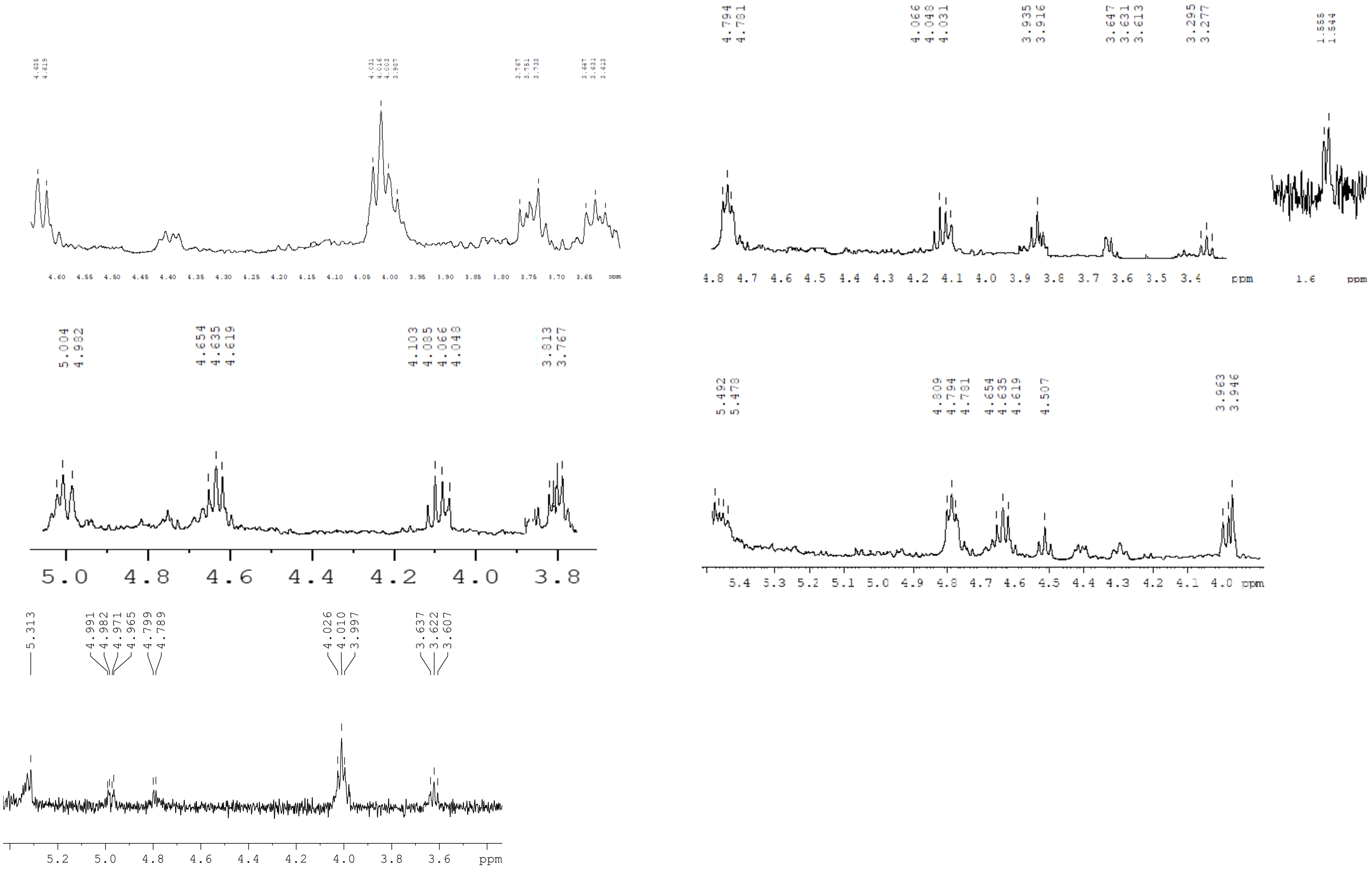

Figure S39. 1D TOCSY (500.12 MHz) spectra of Xyl1, Qui2, Glc3, Glc4, MeGlc5of chilensoside D (**5**) in C_5D_5N/D_2O (4/1)

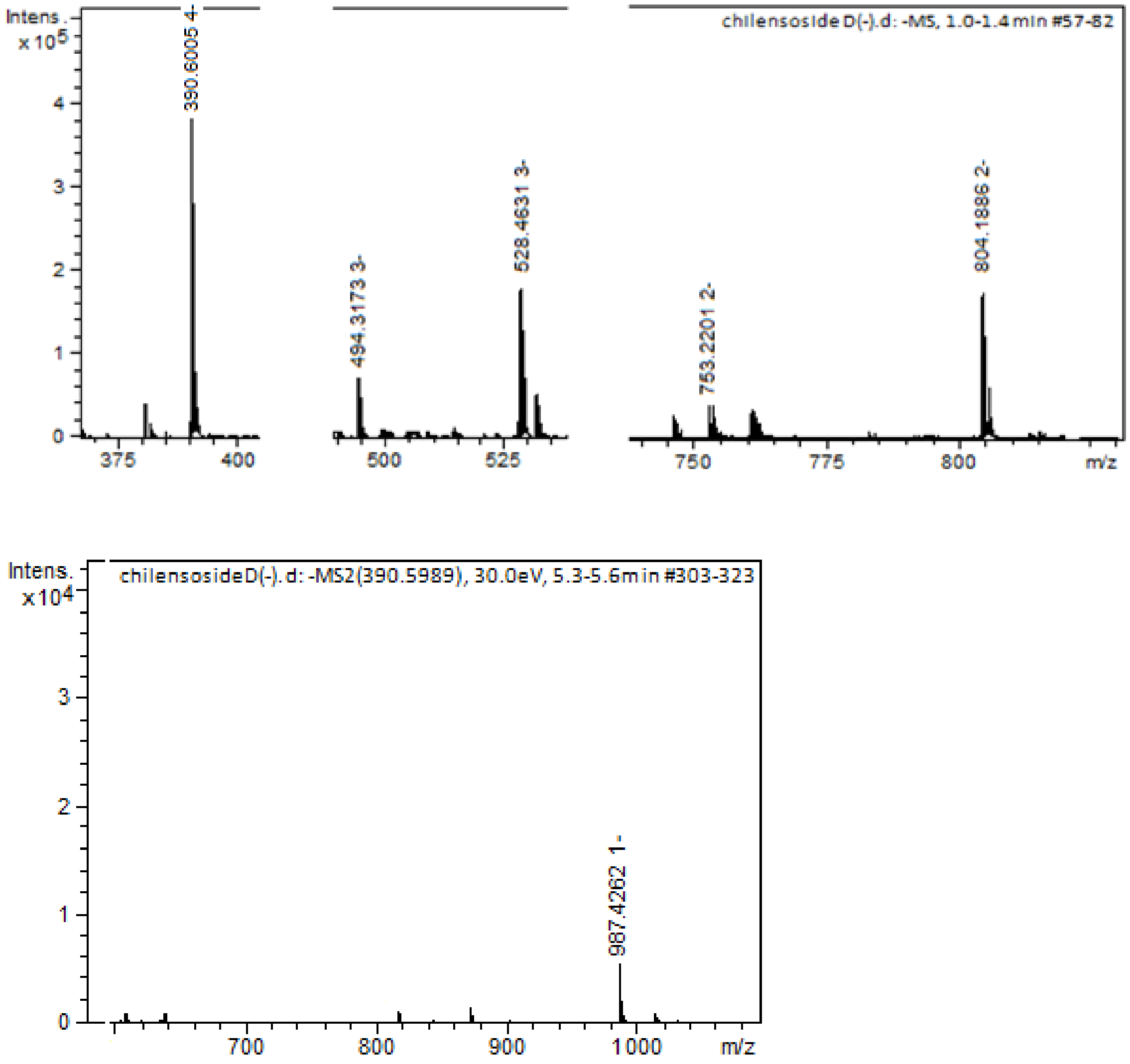

Figure S40. HR-ESI-MS and ESI-MS/MS spectra of chilensoside D (**5**)
